# OsYchF1 suppresses OsEF1Bγ‐mediated ER detoxification to co‐involve the growth‐maintenance trade‐off in rice

**DOI:** 10.1111/tpj.71185

**Published:** 2026-10-30

**Authors:** Liyang Gao, Lulu Yu, Dongyu Chen, Rongfang Li, Yuhe Wang, Yunchuan Liao, Yetao Meng, Qian Li, Ying Miao, Gefei Hao, Rui Miao

**Affiliations:** ^1^ Fujian Provincial Key Laboratory of Plant Functional Biology, College of Life Sciences Fujian Agriculture and Forestry University Fuzhou 350002 P. R. China; ^2^ State Key Laboratory Breeding Base of Green Pesticide and Agricultural Bioengineering, Key Laboratory of Green Pesticide and Agricultural Bioengineering, Ministry of Education Guizhou University Guiyang 550025 P. R. China

**Keywords:** OsEF1Bγ, OsYchF1, post‐translational regulation, osmotic stress, salt stress, redox homeostasis

## Abstract

Translation elongation is a fundamental biological process, yet its direct role in stress adaptation remains unclear. In this study, we uncover a novel regulatory axis in rice involving the unconventional G protein OsYchF1, which gates the detoxification activity of the elongation factor 1Bγ (OsEF1Bγ) in the endoplasmic reticulum. OsEF1Bγ is a subunit of the eEF1B complex and possesses an N‐terminal glutathione S‐transferase (GST) domain. We demonstrate that overexpression of OsEF1Bγ enhances rice tolerance to osmotic and saline stress by lowering malondialdehyde levels, suggesting its role as a positive regulator *via* GST‐mediated detoxification. Conversely, we found that OsYchF1 interacts with OsEF1Bγ across multiple domains. This interaction competitively inhibits the binding of reduced glutathione to OsEF1Bγ by approximately 40‐fold, thereby suppressing its GST activity *in vitro*. Consistently, *OsYchF1* knockout mutants phenocopy OsEF1Bγ overexpressors, displaying enhanced stress tolerance, reduced reactive oxygen species accumulation, and increased antioxidant enzyme activities. Furthermore, we show that stress‐induced downregulation of OsYchF1 acts as a physiological trigger, releasing this molecular brake to enable rapid activation of OsEF1Bγ‐mediated detoxification in response to challenging environments. Collectively, our findings reveal a post‐translational mechanism where OsYchF1 serves as a molecular brake on the ER‐localized detoxification engine of OsEF1Bγ, fine‐tuning abiotic stress adaptation and establishing an unexpected link between the translation machinery and cellular redox homeostasis.

## SUMMARY

The unconventional G protein OsYchF1 acts as a molecular brake on the translation elongation factor OsEF1Bγ, thereby limiting its glutathione‐dependent detoxification process in the endoplasmic reticulum. Stress releases this brake to enhance osmotic and salt tolerance, identifying a tunable target for creating stress‐resilient crops.

## INTRODUCTION

Translation is a highly dynamic and cyclical process comprising four stages: initiation, elongation, termination, and ribosome recycling (Brito Querido et al., 2024). To date, the regulation of elongation has received less attention than others. During elongation, eukaryotic elongation factor 1A (eEF1A) delivers aminoacyl‐tRNA (aa‐tRNA) to the A‐site of the ribosome, forming a ternary complex with aa‐tRNA and GTP (Ejiri, 2002). The eEF1A‐GTP‐aa‐tRNA complex then moves to the 80S ribosome (Dever et al., 2018). Correct codon‐anticodon pairing triggers GTP hydrolysis by eEF1A, inducing a conformational change that facilitates its release from the ribosome, leaving the aa‐tRNA in the A‐site (Janssen & Möller, 1988). The cognate aa‐tRNA is then utilized for peptide bond formation with the peptide‐tRNA in the P‐site. Translocation then occurs, involving the movement of the newly formed peptidyl‐tRNA to the P‐site, the shift of the deacylated tRNA to the E‐site, and the advancement of the mRNA by one codon (Simonović & Steitz, 2009).

After release as an inactive eEF1A‐GDP complex, eEF1A is recycled by the eukaryotic elongation factor 1B (eEF1B) complex, which functions as its guanine nucleotide exchange factor (GEF) (Sasikumar et al., 2012). The eEF1B complex binds to eEF1A‐GDP to promote GDP dissociation and GTP reloading, thereby regenerating the active eEF1A‐GTP complex for subsequent rounds of aa‐tRNA delivery (Bondarchuk et al., 2022). The eEF1B complex in higher plants, according to current nomenclature, comprises three subunits: eEF1Bα, eEF1Bβ, and eEF1Bγ (Le Sourd et al., 2006). The eEF1Bα and eEF1Bβ subunits primarily catalyze GTP/GDP nucleotide exchange, whereas the eEF1Bγ subunit is thought to stabilize the complex and regulate the activity of eEF1Bα and eEF1Bβ (Le Sourd et al., 2006).

The eEF1Bγ subunit, with a molecular mass of 47–52 kDa, consists of two distinct domains: an N‐terminal theta class glutathione S‐transferase (GST) domain and a highly conserved C‐terminal domain (Jeppesen et al., 2003; Koonin et al., 1994). Each domain comprises approximately 200 amino acids (Jeppesen et al., 2003; Koonin et al., 1994). Notably, recombinant rice eEF1Bγ protein exhibits a measurable GST activity (Kobayashi et al., 2001). Glutathione S‐transferases (GSTs) are well known for catalyzing the conjugation of glutathione to a wide range of hydrophobic and electrophilic compounds, thereby facilitating their detoxification and excretion (Stoian et al., 2025; Sun et al., 2025).

The function of *eEF1Bγ* has predominantly been investigated using a loss‐of‐function approach. In yeast, the deletion of *eEF1Bγ* has been shown to inhibit the accumulation of tomato bushy stunt virus (TBSV) RNA (Sasvari et al., 2011), which is consistent with the broader reliance of RNA viruses on host translation factors for replication (Noueiry & Ahlquist, 2003). Similarly, CRISPR/Cas9‐mediated knockout of *eEF1Bγ* in *Nicotiana benthamiana* reduces the accumulation of the tobacco etch virus (TEV) (Kang et al., 2025). Beyond viral infection, impairment of *eEF1Bγ* also compromises growth and development as observed in Arabidopsis (Lohmann et al., 2023). Additionally, under heat stress, small heat shock proteins (sHSPs) and the chaperone HSP101 help to maintain the solubility of eEF1B subunits and protect translation factors (McLoughlin et al., 2016). Despite these insights, the precise molecular mechanisms underlying the detoxification function of eEF1Bγ in the rice response to abiotic stress remain largely unexplored.

On the other hand, the well‐characterized unconventional G protein OsYchF1, distinct from canonical heterotrimeric and small G proteins, has been identified as a negative regulator of plant stress responses and also plays a fundamental role in protein biosynthesis (Lin et al., 2023; Luo et al., 2022). Supporting this, *Arabidopsis thaliana YchF1* (*AtYchF1*) T‐DNA insertion mutants exhibit enhanced tolerance to the bacterial pathogen *Pseudomonas syringae pv*. tomato DC3000 (*Pst* DC3000), whereas *OsYchF1* overexpression lines display reduced resistance to the same pathogen (Cheung et al., 2008). Furthermore, the overexpression of either *OsYchF1* or *AtYchF1* in transgenic *A. thaliana* results in increased sensitivity to the oxidative stress induced by high salinity (Cheung et al., 2013). Beyond its role in stress signaling, YchF mediates translation by associating with the ribosome (Becker et al., 2012; Tomar et al., 2011; Yu et al., 2025). For instance, *E. coli* YchF binds to the prokaryotic translation initiation factor 3 (IF3) to balance protein synthesis and stress responses. Meanwhile, the human YchF homolog hOLA1 interacts with the eukaryotic initiation factor 2 (eIF2) to inhibit protein synthesis (Chen et al., 2015; Landwehr et al., 2021).

Despite the known structural features of eEF1Bγ, such as its N‐terminal GST domain, its precise role in orchestrating abiotic stress responses in rice remains largely enigmatic. Given the unconventional nature of the G protein OsYchF1 and its known interaction with translation machinery, we hypothesized that OsYchF1 functions as a molecular rheostat that fine‐tunes rice stress adaptation by directly modulating the ER‐localized GST activity of OsEF1Bγ. To test this, we employed a two‐pronged genetic and biochemical strategy. First, we generated overexpression lines of *OsEF1Bγ* to evaluate whether enhancing this “detoxification engine” provides a gain‐of‐function benefit under osmotic and salinity stress. Complementarily, we utilized CRISPR/Cas9‐mediated knockout lines of *OsYchF1* to determine if removing this inhibitory “brake” results in a similar increase in stress resilience. By integrating these genetic approaches with thermodynamic binding studies and structural docking, we aim to dissect the regulatory circuit that balances rice growth and stress defense at the level of the translational apparatus.

## RESULTS

### Overexpression of *OsEF1Bγ* confers rice tolerance to osmotic and salt stresses

To explore the biological function of the OsEF1Bγ subunit of the eEF1B complex in rice, three independent *OsEF1Bγ* overexpression lines (OE#1, OE#3, and OE#5) were generated and driven by the cauliflower mosaic virus (CaMV) 35S promoter in a Nipponbare background. First, we analyzed the transcript levels of *OsEF1Bγ* in the overexpression transgenic rice plants using quantitative real‐time PCR (qRT‐PCR). qRT‐PCR showed that *OsEF1Bγ* transcript levels were significantly higher in the 4‐week‐old *OsEF1Bγ* overexpression lines (OE#1, OE#3, and OE#5) than in the wild‐type (WT) plants (Figure 1B). Second, qRT‐PCR exhibited that the *OsEF1Bγ* transcript level was highest in the roots of WT plants (Figure 1C), suggesting that *OsEF1Bγ* is predominantly expressed in the rice roots.

**Figure 1:**
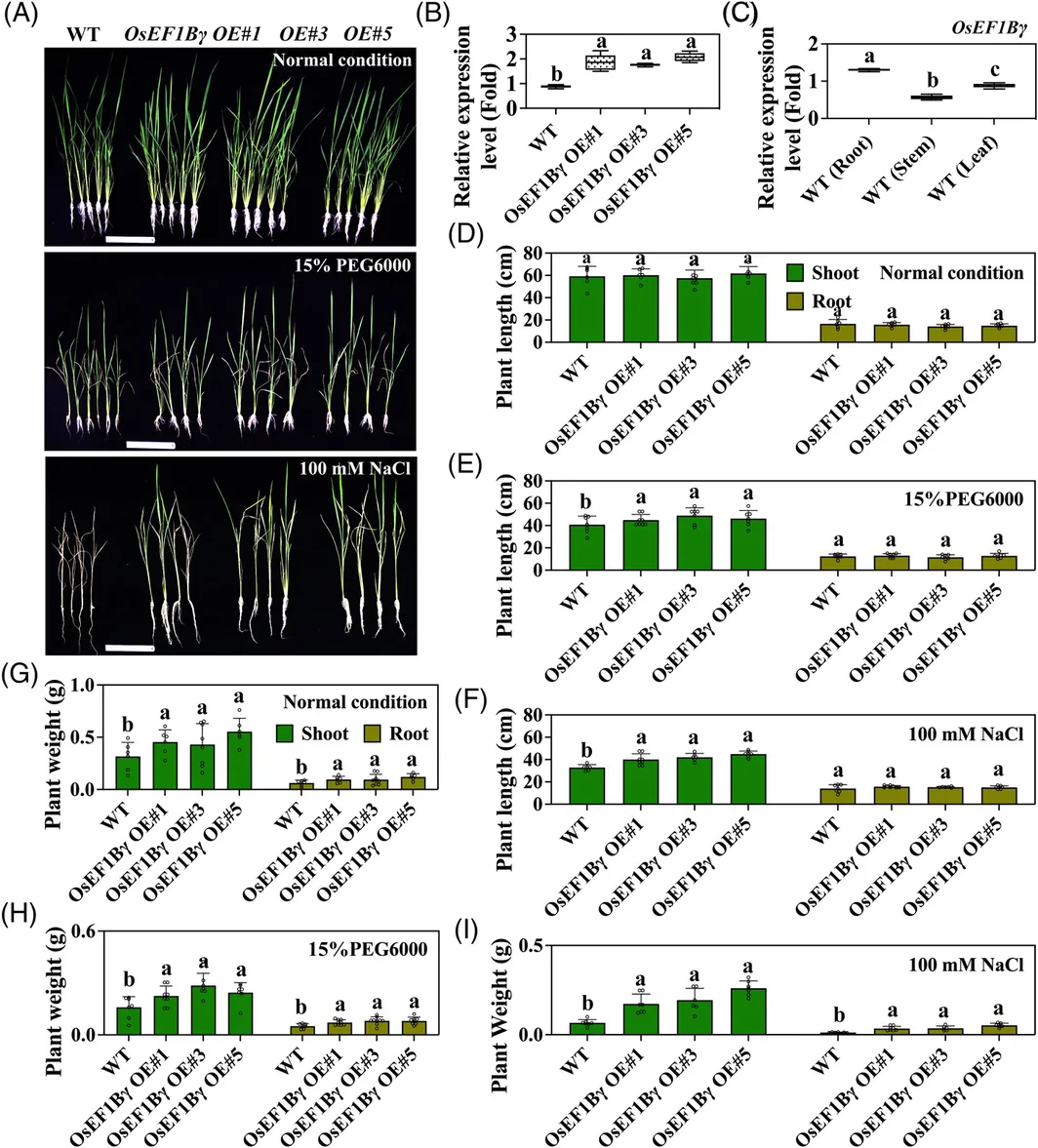
*OsEF1Bγ* overexpression confers rice tolerance against osmotic and salt stresses. (A) Representative phenotypes of 8‐week‐old wild‐type (WT) Nipponbare (Nip) and *OsEF1Bγ* overexpression transgenic rice lines OE#1, OE#3, and OE#5 using Nip as the background in normal conditions, 15% PEG6000 caused osmotic stress and 100 mM high salinity stress. (B) The relative expression of *OsEF1Bγ* in the leaves of 4‐week‐old WT, *OsEF1Bγ* OE#1, OE#3, and OE#5 plants was investigated using qRT‐PCR. (C) The relative expression of *OsEF1Bγ* in the roots, stems, and leaves of WT plants was analyzed by qRT‐PCR. (D–F) The plant length of 8‐week‐old WT, *OsEF1Bγ* OE#1, OE#3, and OE#5 plants in normal conditions (No significant differences were observed), 15% PEG6000 caused osmotic stress and 100 mM high salinity stress conditions. (G–I) The shoot and root dry weight of 8‐week‐old WT, *OsEF1Bγ* OE#1, OE#3, and OE#5 plants in normal conditions, 15% PEG6000 caused osmotic stress and 100 mM high salinity stress conditions. Scale bars = 20 cm. Error bars indicate the SD; *n* ≥ 4, and each experiment was repeated at least three times. Different letters above the bars indicate statistically significant differences (*P* < 0.05) as determined by Tukey's HSD test.

To determine the role of *OsEF1Bγ* in rice responses to environmental stimuli, we observed the phenotype of gain‐of‐function *OsEF1Bγ* transgenic rice plants in liquid media under normal conditions, as well as under osmotic stress caused by polyethylene glycol (PEG6000) and high salinity conditions (Figure 1A). Under normal conditions, there were no notable differences in plant length between *OsEF1Bγ* overexpression lines and corresponding WT rice plants at the vegetative stage (Figure 1D). However, the 8‐week‐old *OsEF1Bγ* overexpression lines exhibit noticeably higher shoot and root biomass than WT plants, indicating that *OsEF1Bγ* overexpression boosts overall vegetative vigor and dry matter accumulation (Figure 1G). An increase in biomass without a corresponding increase in height is commonly observed when genetic modifications affect lateral growth, tiller production, or organ thickness rather than internode elongation.

Following treatment with 15% PEG6000 and 100 mM sodium chloride (NaCl), the dry weights of both the shoots and roots were greater in the 8‐week‐old *OsEF1Bγ* overexpression lines (Figure 1H,I), and the shoot length was also notably longer in the 8‐week‐old *OsEF1Bγ* overexpression lines compared to WT plants (Figure 1E,F). Overall, these results demonstrate that *OsEF1Bγ* overexpression enhances rice growth under normal conditions and confers resistance to osmotic and salt stresses during the vegetative stage.

### OsYchF1 binds to OsEF1Bγ in the endoplasmic reticulum

To identify potential translation‐related interactors of OsYchF1 on a proteomic scale, we conducted immunoprecipitation‐mass spectrometry (IP‐MS) using FLAG‐tagged OsYchF1. Among the total proteins co‐purified, we curated a subset of translation‐related candidates, which are summarized in Table S1. After removing redundant isoforms and consolidating entries by gene locus, these data represented approximately 30 distinct translation‐related protein groups. The identified proteins covered multiple functional categories of the translational machinery, including initiation factors (e.g., eukaryotic initiation factor 4A (eIF4A) isoforms and eIF5), elongation factors (e.g., eEF1A, eEF1Bγ, eEF2, and chloroplastic EF‐Tu), and a wide array of ribosomal proteins (including 40S subunits and 60S subunits). Notably, eEF1A and eIF4A were detected with high confidence, yielding 12 and 14 unique peptides, respectively, with favorable Rank scores of ~9.1 and ~5.3–5.5.

Importantly, our candidate of interest, OsEF1Bγ, was confidently identified with four unique peptides and a Rank score of approximately 6.1. Moreover, OsEF1Bγ is a core component of the eukaryotic translation elongation complex, which is functionally closely related to the reported translational regulatory role of OsYchF1. Collectively, these IP‐MS data reveal a robust and diverse interaction network between OsYchF1 and core components of the translation apparatus, and they provide the direct proteomic evidence that prompted us to select OsEF1Bγ for further validation of the OsYchF1—OsEF1Bγ interaction.

To verify the interaction between the translation elongation factor OsEF1Bγ and the unconventional G protein OsYchF1, we first performed Firefly Luciferase Complementation Imaging (LCI) assays. Plasmids expressing *OsEF1Bγ* fused to the C‐terminal fragment of luciferase (cLuc) and *OsYchF1* fused to the N‐terminal fragment (nLuc) were constructed. These constructs—N‐terminally tagged cLuc‐OsEF1Bγ and C‐terminally tagged OsYchF1‐nLuc—were transiently co‐expressed in tobacco (*Nicotiana benthamiana*) plant leaves. A strong luciferase signal was detected, indicating an *in vivo* interaction, while all negative controls showed only background luminescence (Figure 2A).

**Figure 2:**
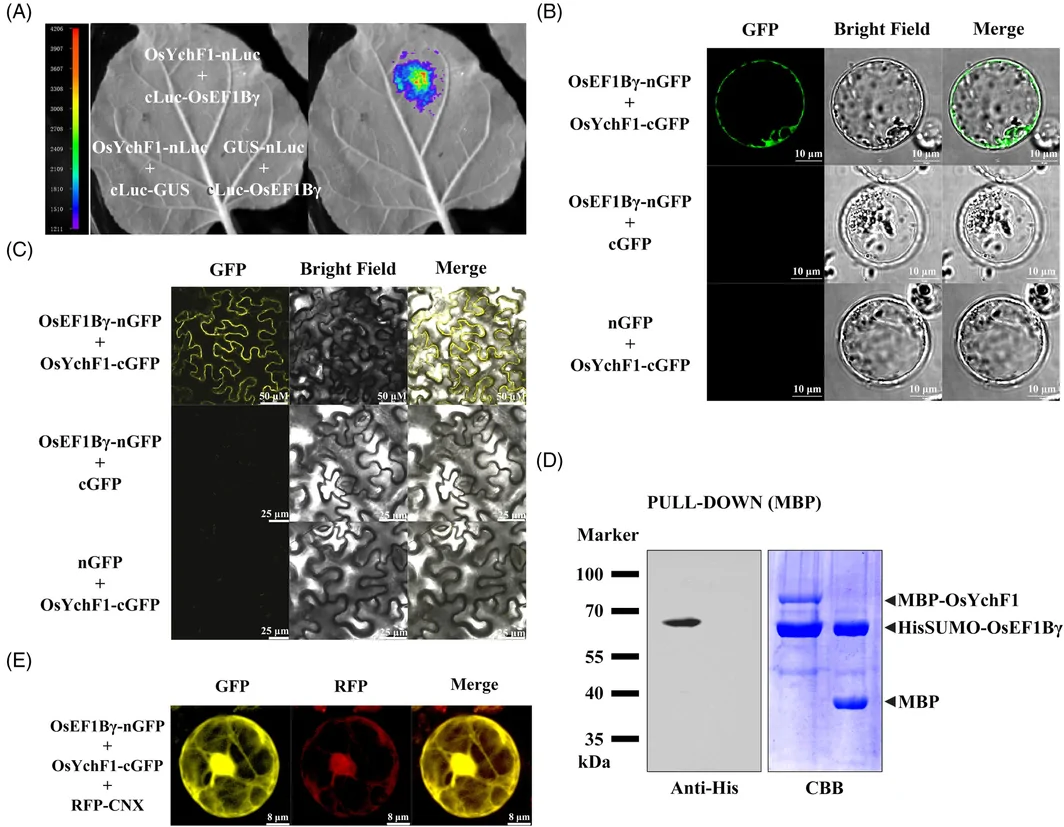
OsEF1Bγ binds to OsYchF1 in the endoplasmic reticulum. (A) Luciferase complementation imaging (LCI) assays were conducted in tobacco (*Nicotiana benthamiana*) plants co‐expressing the indicated constructs for 48 h. A strong luminescent signal confirmed the interaction between OsYchF1‐nLuc and cLuc‐OsEF1Bγ. Negative controls (GUS‐nLuc/cLuc‐OsEF1Bγ and OsYchF1‐nLuc/cLuc‐GUS) showed only background signal. (B, C) Bimolecular fluorescence complementation (BiFC) assays show the *in vivo* interaction between OsEF1Bγ‐nGFP and OsYchF1‐cGFP in rice leaf protoplasts (B) and *N. benthamiana* leaf epidermal cells (C). (D) Recombinant MBP‐OsYchF1 (or MBP alone) was immobilized on Maltose‐Binding Protein beads and incubated with recombinant HisSUMO‐OsEF1Bγ. After extensive washing, bound proteins were detected by anti‐His antibody (or Coomassie staining). OsEF1Bγ was specifically retained by MBP‐OsYchF1, but not by MBP alone or by the beads only, indicating a direct physical interaction. CBB means Coomassie Brilliant Blue. (E) Subcellular localization of the interaction. A BiFC assay in rice protoplasts co‐expressing OsYchF1‐cGFP, OsEF1Bγ‐nGFP, and the endoplasmic reticulum (ER) marker RFP‐CNX (ER‐mCherry). The merged image shows clear co‐localization of the BiFC signal (yellow) with the ER marker (red), demonstrating that the OsYchF1‐OsEF1Bγ interaction occurs specifically in the endoplasmic reticulum. Scale bars = 10 μm. Cells were imaged using confocal microscopy.

To confirm the *in vivo* interaction between OsEF1Bγ and OsYchF1, Bimolecular Fluorescence Complementation (BiFC) assays were performed. We constructed plasmids expressing *OsEF1Bγ* fused to the N‐terminal fragment of GFP (nGFP) and *OsYchF1* fused to the C‐terminal fragment (cGFP). These constructs were co‐transformed into rice leaf protoplasts. After an overnight incubation, a clear green fluorescent protein (GFP) fluorescence signal was observed, confirming that OsEF1Bγ binds to OsYchF1 (Figure 2B). This interaction was then validated by co‐expressing the same constructs in *N. benthamiana* leaves, where distinct GFP signals were also detected (Figure 2C). No fluorescence was observed in negative control combinations (Figure 2B,C).

To corroborate the intracellular interaction observed by LCI and BiFC, we performed an *in vitro* pull‐down assay. The purified recombinant HisSUMO‐OsEF1Bγ was incubated with MBP‐OsYchF1 or the control MBP protein. As shown in Figure 2D, HisSUMO‐OsEF1Bγ was specifically co‐precipitated with MBP‐OsYchF1, but not with MBP alone. These results provide biochemical evidence for a direct physical interaction between OsYchF1 and OsEF1Bγ, excluding the possibility that the interaction observed in planta is mediated by other bridging factors.

Interestingly, following the co‐expression of OsEF1Bγ‐nGFP and OsYchF1‐cGFP, we observed GFP signals in the cytoplasm (Figure 2B). As OsEF1Bγ is a translation‐related elongation factor, we speculate that OsYchF1 might interact with OsEF1Bγ within the cytosolic endoplasmic reticulum (ER). To prove the subcellular localization of the ER, we co‐expressed OsEF1Bγ‐nGFP and OsYchF1‐cGFP alongside an ER membrane protein marker (RFP‐CNX, calnexin) in rice leaf protoplasts. The OsEF1Bγ‐OsYchF1 GFP fluorescence signal actually overlapped with the tubular and mesh‐like pattern of the ER labeled by RFP‐CNX, validating that OsEF1Bγ is at least partially co‐localized with OsYchF1 in the ER (Figure 2E).

### All domains of OsEF1Bγ and OsYchF1 are implicated in their interaction

To define the essential interacting regions between OsEF1Bγ and OsYchF1 and delineate their structural basis, a series of truncated derivatives was constructed based on their domain architecture. For OsYchF1, these truncated derivatives included the N‐terminal G domain (amino acid (aa) 1–128), the inserted α‐helical domain (aa 141–199), the C‐terminal TGS domain (aa 303–394), and a combined G‐helical “No TGS” domain (aa 1–302) (Figure 3A). For OsEF1Bγ, the truncated derivatives comprised its N‐terminal GST domain (aa 1–208) and its conserved C‐terminal domain (aa 240–416) (Figure 3A).

**Figure 3:**
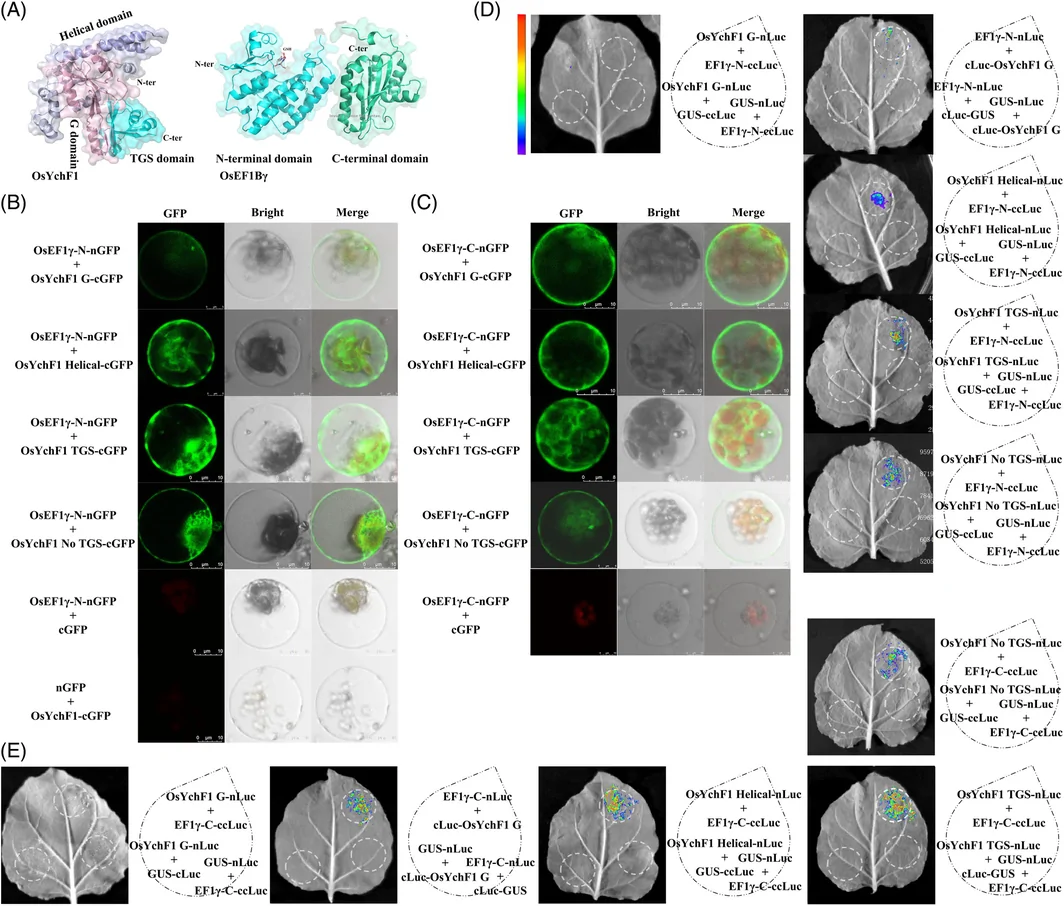
All domains of OsEF1Bγ and OsYchF1 are implicated in their interaction. (A) Representative cartoon images showing the OsYchF1 and OsEF1Bγ. OsYchF1 consists of a G domain, an α‐helical domain, and a TGS domain from the N‐terminus to the C‐terminus; OsEF1Bγ is divided into an N‐terminal GST‐like domain and a C‐terminal domain. Glutathione (GSH) is depicted as a stick. (B and C) Mapping of the domains involved in the OsEF1Bγ‐OsYchF1 interaction using BiFC assays. The N‐terminal OsEF1Bγ‐N‐nGFP or C‐terminal OsEF1Bγ‐C‐nGFP was co‐expressed with OsYchF1‐cGFP truncated versions, including the G domain, helical domain, TGS domain, and No TGS domain. Negative controls of OsEF1Bγ‐N‐nGFP with cGFP and nGFP with OsYchF1‐cGFP (B), as well as OsEF1Bγ‐C‐nGFP with cGFP (C), revealed only background signals. (D) LCI assays were used to detect the interaction of the N‐terminal OsEF1Bγ or C‐terminal OsEF1Bγ with OsYchF1 truncated versions (G domain, Helical domain, TGS domain, or “No TGS” domain). The OsEF1Bγ N‐terminal domain (D) and C‐terminal domain (E) were fused with the C‐terminal fragment of luciferase (ccLuc), while the different domains of OsYchF1 (G domain, helical domain, TGS domain, and No TGS domain) were fused with the N‐terminal fragment of luciferase (nLuc). The LCI images of *Nicotiana benthamiana* leaves co‐infiltrated with the agrobacterial strain GV3101 containing the indicated fusion proteins.

The interaction between these individual domains was systematically investigated *in vivo* using BiFC and LCI assays. BiFC assays revealed that each isolated domain of OsYchF1 (G, α‐helical, TGS, and No TGS) was capable of generating a positive GFP signal when paired with either the N‐terminal GST domain (Figure 3B) or the C‐terminal domain (Figure 3C) of OsEF1Bγ (Figure S1). All tested pairwise domain combinations yielded fluorescence significantly above background levels, although the signal intensity varied among different pairs, suggesting possible differences in binding affinity or conformational stability (Figure 3B,C). The fluorescence was primarily localized to the cytoplasm, consistent with the known subcellular distribution of both intact proteins. By contrast, co‐expression of the negative controls, comprising OsEF1Bγ‐N‐nGFP and OsEF1Bγ‐C‐nGFP with the cGFP vector, or OsYchF1‐cGFP with the nGFP vector, failed to produce a clear GFP signal in the rice leaf protoplasts (Figure 3B,C).

These findings were further substantiated by LCI assays. The LCI assays showed that the OsEF1Bγ‐N‐nLuc and OsEF1Bγ‐C‐nLuc fusion proteins accumulated to a similar level of luminescence signal when co‐expressed with OsYchF1 (Helical domain)‐ccLuc, OsYchF1 (TGS domain)‐ccLuc, or OsYchF1 (No TGS domain)‐ccLuc (Figure 3D,E). Nevertheless, both C‐terminally tagged OsEF1Bγ‐N‐nLuc and OsEF1Bγ‐C‐nLuc co‐expressed with C‐terminally tagged OsYchF1 (G domain)‐ccLuc showed merely background level luciferase activity (Figure 3D,E). This result was in contrast to our BiFC data (Figure 3B,C) and suggested that the C‐terminal ccLuc fusion tag on the OsYchF1 G domain may sterically hinder the interaction interface. Indeed, when we used an N‐terminal tag (cLuc‐OsYchF1 G domain) to circumvent this potential steric clash, clear luminescence signals were observed with both OsEF1Bγ‐N‐nLuc and OsEF1Bγ‐C‐nLuc, confirming that the C‐terminal region of OsYchF1 G domain is required for the interaction of OsYchF1 G domain with OsEF1Bγ truncated derivatives (Figure 3D,E).

Collectively, BiFC and LCI results verified that each defined structural domain of OsYchF1 can independently interact with the two bi‐terminal domains of OsEF1Bγ. These multiple contact regions suggest that the OsEF1Bγ‐OsYchF1 complex is stabilized cooperatively across its entire structure, which may be crucial for the coordinated functional roles of these proteins in cellular processes.

### OsYchF1 inhibits OsEF1Bγ GST activity *via* GSH‐access restriction

Previous studies have demonstrated that the N‐terminal domain of OsEF1Bγ exhibits low but detectable GST activity, which plays a key role in cellular detoxification (Kobayashi et al., 2001; Lohmann et al., 2023; Tshabalala et al., 2016). To improve our understanding of the OsEF1Bγ and OsYchF1 complex, we attempted to evaluate the enzyme activity of OsEF1Bγ in conjunction with OsYchF1 *in vitro*. Initially, we performed structural sequence alignments of OsEF1Bγ with its homologs from *Oryza sativa*, *A. thaliana*, and *Saccharomyces cerevisiae* using Jalview version 2.8.2 (https://www.jalview.org/) (Figure S2). This analysis revealed that OsEF1Bγ shares 83.3% amino acid sequence identity with OsEF1Bγ‐1 (BAA34206.1) in rice, 72.8% with AtEF1Bγ (AT1G57720) in Arabidopsis, and 31.7% with ScEF1Bγ (NP_015277.1) in yeast. Subsequent phylogenetic analysis confirmed that OsEF1Bγ has a higher degree of sequence identity with other plant homologs than with yeast homologs (Figure S3).

Notably, most GSTs form dimers, and dimerization has also been reported for EF1Bγ from *Saccharomyces cerevisiae* (Jeppesen et al., 2003). Previous studies on yeast eEF1Bγ (Tef3p) demonstrated that the isolated N‐terminal GST‐like domain 1 fragment (residues 1–219) behaved as a monomer during gel filtration chromatography. In contrast, the slightly extended domain 1′ fragment (residues 1–242) eluted at a position consistent with dimer formation, suggesting that the short extension adjacent to the GST‐like core contributes to stable dimerization of eEF1Bγ (Le Sourd et al., 2006). This observation aligns with the general behavior of cytosolic GSTs, which commonly function as dimeric proteins, with dimer assembly contributing to the formation and stabilization of the ligand‐binding environment (Wallace & Dirr, 1999). Therefore, we used AlphaFold3 to predict the dimeric structure of OsEF1Bγ and consequently performed molecular docking with GSH using this model. The top‐ranked predicted model (interface predicted Template Modeling (ipTM) score 0.56, predicted Template Modeling (pTM) score 0.57, and ranking score 0.62) is presented in Figure 4A. The ipTM (0.56) and pTM (0.57) scores fall within the moderate range (0.5–0.7) for heterodimer prediction. An average interchain PAE of 19.83 Å indicates that the positioning of chains A and B relative to each other is moderately resolved. The model yields a mean pLDDT score of 82.43, with 82.46 for chain A and 82.39 for chain B, demonstrating good confidence in the local folding of the polypeptide chains. After that, AutoDock Vina was used to predict the binding position of reduced glutathione (GSH), localizing it to a pocket‐like binding site on OsEF1Bγ (Figure 4A,B).

**Figure 4:**
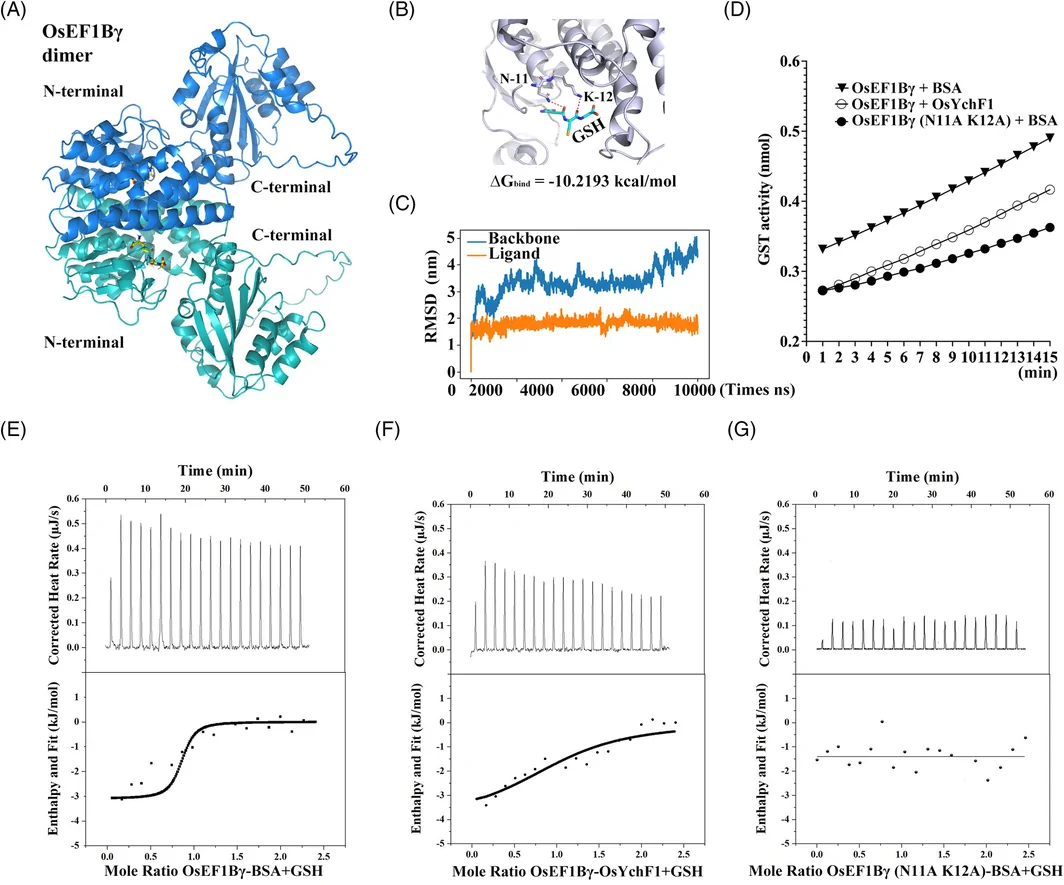
OsYchF1 inhibits the GST activity of OsEF1Bγ by reducing its binding affinity to GSH. (A) Schematic structure of dimeric OsEF1Bγ bound to glutathione (GSH). The N‐terminal domain is shown in dark green, and the C‐terminal domain in light green. (B) Molecular dynamics simulation‐derived binding mode of GSH (stick, cyan) with OsEF1Bγ (cartoon, light blue). Visualization was performed using PyMOL. (C) Root‐mean‐square deviation (RMSD) analysis from Amber 24 simulations, processed using Python, illuminating RMSD fluctuations in the GSH ligand and the OsEF1Bγ backbone over time. (D) Time course of intrinsic GST activity of purified OsEF1Bγ over 15 min. The GST activity was measured in the presence of OsYchF1 or bovine serum albumin (BSA) as a control. The molar ratio of OsYchF1 to OsEF1Bγ was approximately 1–2. Error bars represent standard deviation (*n* = 4). (E) Isothermal titration calorimetry (ITC) analysis of the interaction of 60 μM purified OsEF1Bγ with 500 μM GSH, with 60 μM BSA included as a control. (F) ITC analysis of 60 μM purified OsEF1Bγ binding to 500 μM GSH in the presence of 60 μM OsYchF1. (G) ITC analysis of 60 μM purified OsEF1Bγ (N11A K12A) binding to 500 μM GSH, with 60 μM BSA included as a control. All ITC experiments were performed at 25°C and repeated twice with consistent results. The upper panels show the raw heat signals over time during titration, and the lower panels show the integrated binding isotherms.

Importantly, molecular dynamics (MD) simulations were employed to validate the binding stability of the GSH ligand within the OsEF1Bγ binding pocket. Following a 100 ns trajectory, conformational analysis *via* structural superimposition confirmed the formation of a robust complex. As illustrated in Figure 4C, the root‐mean‐square deviation (RMSD) for the OsEF1Bγ receptor and the GSH ligand equilibrated at approximately 7.89 Å and 1.87 Å, respectively. The thermodynamic profile, detailed in Figure 4C, revealed an overall binding free energy (∆G_bind_) of −10.2193 kcal/mol, suggesting a highly favorable and spontaneous interaction (Figure 4B). This high affinity is primarily driven by specific polar interactions; notably, GSH established a stable hydrogen‐bonding network with the Asn‐11 and Lys‐12 residues of the OsEF1Bγ protein (Figure 4B). The two predicted binding residues (Asn‐11 and Lys‐12) in OsEF1Bγ are highly conserved in rice and Arabidopsis, but not in yeast (Figure S3).

To investigate the interaction mechanism and confirm the prediction further, we constructed expression vectors for the recombinant production and subsequent purification of OsYchF1, OsEF1Bγ, and a double point‐mutated version of OsEF1Bγ (N11A, K12A) (Figure S5). *In vitro* biochemical assays revealed that OsEF1Bγ exhibits measurable GST activity within a 15‐min reaction period (Figure 4D). Intriguingly, the addition of OsYchF1 inhibited the enzyme activity, suggesting that OsYchF1 acts as a negative regulator that restricts the catalytic efficiency or substrate accessibility of OsEF1Bγ (Figure 4D). Therefore, OsYchF1 not only physically associates with OsEF1Bγ but also represses its enzymatic activity *in vitro*. Moreover, the OsEF1Bγ (N11A, K12A) mutant displayed notably decreased GST activity compared with the WT protein, indicating that these two amino acid residues (Asn‐11 and Lys‐12) are necessary for the OsEF1Bγ enzyme activity (Figure 4D).

### Thermodynamic insights into the negative impact of OsYchF1 on the OsEF1Bγ

To gain mechanistic insight into the inhibition, the binding affinity between OsEF1Bγ and reduced glutathione (GSH) was further quantified using isothermal titration calorimetry (ITC). ITC was employed to determine the thermodynamic parameters and binding equilibrium (K_*D*_) by monitoring the entropic and enthalpic changes associated with the protein‐ligand interaction (Miao et al., 2019). This approach provided a direct measurement of the binding stoichiometry and affinity, complementing the MD simulation and enzymatic inhibition data.

Thermodynamic data revealed that 60 μM OsEF1Bγ binds tightly to 500 μM GSH at micromolar concentrations, with a K_*D*_ value of 0.203 ± 5.607 μM (Figure 4E; Table 1). The OsEF1Bγ–GSH interaction demonstrated an unusual endothermic association, driven by a substantial entropic change (−T∆S = −35.092 kJ/mol) and a modest enthalpic change (∆H = −3.099 ± 6.947 kJ/mol) (Table 1). These results suggest that the binding process is governed predominantly by the release of ordered water molecules or significant conformational rearrangements upon complex formation, while the small enthalpy change highlights the role of hydrogen bonding in stabilizing the interaction. The binding isotherm for this interaction was fitted to a single‐site model to derive the thermodynamic parameters listed in Table 1. The stoichiometric value (N) was determined to be 0.812 ± 0.259 for GSH, indicating that one OsEF1Bγ molecule binds to one GSH ligand only (Table 1).

**Table 1:** ITC binding constants and thermodynamic parameters for interactions of OsEF1Bγ with GSH in the presence of BSA and OsYchF1

	*K*_D_ (μM)	*N*	Δ*H*	–*T*Δ*S*	Δ*G*
			(kcal/mol)	(kcal/mol)	(kcal/mol)
OsEF1Bγ‐BSA with GSH	0.203 ± 5.607	0.812 ± 0.259	−3.099 ± 6.947	−35.092	−38.191
OsEF1Bγ‐OsYchF1 with GSH	8.31 ± 20.023	1.089 ± 0.492	−3.992 ± 21.83	−25.009	−29.001
OsEF1Bγ (N11A K12A)‐BSA with GSH	N.D.	N.D.	N.D.	N.D.	N.D.

Experiments were carried out at 25°C, and each value is the mean of three independent titrations. N.D., not detected; *K*_D_, dissociation constant; *n* value, number of binding sites (*n* = ligand/receptor); Δ*H* (enthalpy change), Δ*S* (entropy change), and Δ*G* (Gibbs free energy). The binding entropy and enthalpy determine ligand binding. Positive is unfavorable and negative is favorable (Tzeng & Kalodimos, 2009; Tzeng & Kalodimos, 2012).

Crucially, the effect of OsYchF1 on the binding between OsEF1Bγ and GSH was investigated by re‐determining the thermodynamic parameters using ITC and fitting them to a simple one‐site binding model. GSH was titrated into a preformed OsEF1Bγ–OsYchF1 complex at a 1:1 molar ratio. The ITC profile revealed that the binding remained endothermic with a favorable entropic contribution (–TΔS = −25.009 kJ/mol) and a small enthalpic change (ΔH = −3.992 ± 21.83 kJ/mol) (Figure 4F and Table 1). Notably, the dissociation constant increased markedly to K_*D*_ = 8.31 ± 20.023 μM, representing an approximately 40‐fold weakening compared to GSH binding to OsEF1Bγ in the presence of the BSA control (K_*D*_ = 0.203 ± 5.607 μM) (Table 1). Overall, the thermodynamic data strongly suggest that OsYchF1 directly impedes GSH binding to OsEF1Bγ.

Furthermore, the thermodynamic analysis uncovered that the OsEF1Bγ (N11A, K12A) mutant had lost the ability to bind GSH entirely (Figure 4G and Table 1). No detectable signal was observed when 500 μM GSH was titrated into the 60 μM OsEF1Bγ (N11A, K12A) double mutant protein (Figure 4G and Table 1). These results confirm that residues Asn‐11 and Lys‐12 of OsEF1Bγ are indispensable for GSH binding and demonstrate that this binding deficiency accounts for the reduced GST enzyme activity of the OsEF1Bγ (N11A, K12A) mutant.

### *OsYchF1* knockout mutants show marked tolerance to osmotic and high salinity stress

To examine the role of *OsYchF1* in the rice response to osmotic and high salinity stress and to test if impaired *OsYchF1* relieves *OsEF1Bγ*‐mediated effects, we created three independent *OsYchF1* knockout mutants in the Nipponbare background using the CRISPR‐Cas9 system as a tool (hereafter denoted *Osychf1‐1*, *Osychf1‐3*, and *Osychf1‐5*) (Figure S4A–C). First, we analyzed the transcript levels of *OsYchF1* in the knockout mutants using quantitative real‐time PCR (qRT‐PCR). The abundance of *OsYchF1* mRNA was dramatically lower in 4‐week‐old mutants (*Osychf1‐1*, *Osychf1‐3*, and *Osychf1‐5*) than in the corresponding WT Nipponbare (Figure 5D). Second, 4‐week‐old WT, *Osychf1‐1*, and *Osychf1‐5* seedlings, pre‐grown in liquid media, were subjected to osmotic stress by being treated with 15% PEG6000 or to high salinity stress by being treated with 100 mM NaCl for an additional 4 weeks.

**Figure 5:**
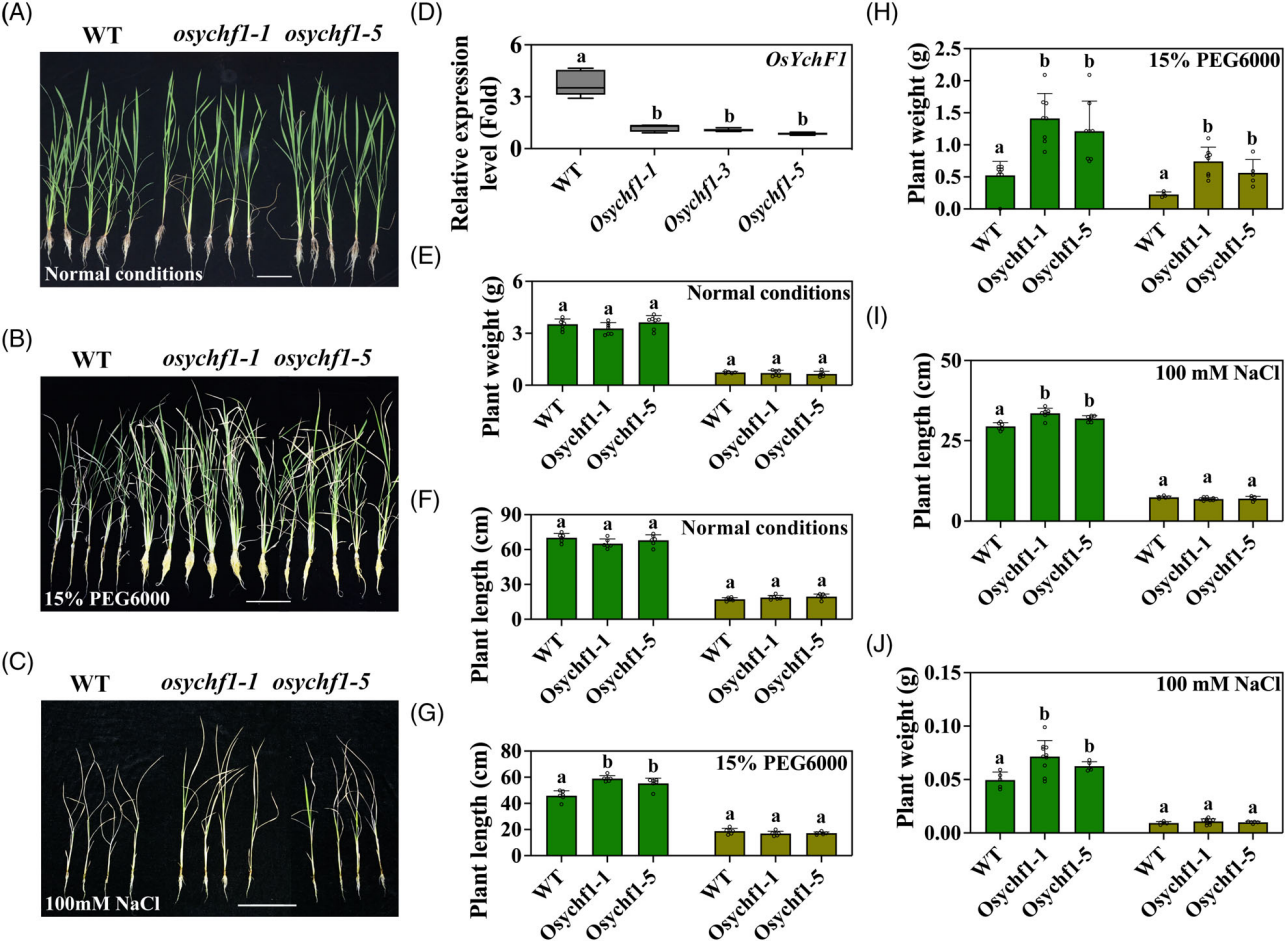
*OsYchF1* knockout mutants confer tolerance to osmotic and high salinity stress in rice. Representative phenotypes of 9‐week‐old WT Nipponbare (Nip) and CRISPR‐Cas9‐based gene‐editing transgenic rice lines *Osychf1‐1* and *Osychf1‐5*, with Nip as the background, are shown under normal conditions (A) and 15% PEG6000 caused osmotic stress (B). (C) Representative phenotypes of 6‐week‐old WT Nip, *Osychf1‐1*, and *Osychf1‐5* are shown under 100 mM NaCl treatment. (D) The relative expression of *OsYchF1* in WT, *Osychf1‐1*, and *Osychf1‐5* leaves was investigated using qRT‐PCR. (E) Shoot and root dry weight of 8‐week‐old WT, *Osychf1‐1*, and *Osychf1‐5* plants under normal conditions. (F) Plant length of 8‐week‐old WT, *Osychf1‐1*, and *Osychf1‐5* plants under normal conditions. (G) Shoot and root dry weight of 8‐week‐old WT, *OsYchF1‐1*, and *OsYchF1‐5* plants upon osmotic stress caused by 15% PEG6000. (I) Plant length of 8‐week‐old WT, *Osychf1‐1*, and *Osychf1‐5* plants upon osmotic stress caused by 15% PEG6000. (J) Shoot and root dry weight of 6‐week‐old WT, *Osychf1‐1*, and *Osychf1‐5* plants after 100 mM NaCl treatment. (H) Plant length of 6‐week‐old WT, *Osychf1‐1*, and *Osychf1‐5* plants after 100 mM NaCl treatment. Error bars indicate the SD; *n* ≥ 5, and each experiment was repeated at least three times. Scale bars = 10 cm. Different letters above the bars indicate statistically significant differences (*P* < 0.05) as determined by Tukey's HSD test.

Subsequently, no significant differences in morphological growth or biomass accumulation were observed between 9‐week‐old WT and *OsYchF1* knockout mutants under normal conditions (Figure 5A,E,F). However, upon 15% PEG6000 or 100 mM NaCl treatment, the *Osychf1‐1* and *Osychf1‐5* mutants accumulated noticeably greater dry biomass in both shoots and roots than WT plants (Figure 5B,C,H,J). Moreover, the mutants developed taller shoots under stress, while root elongation remained comparable to that of WT (Figure 5B,C,G,I). As the initial plant biomass was equivalent across lines before treatment, these results collectively indicate that the loss of *OsYchF1* function confers a growth advantage in response to abiotic stress, suggesting that it acts as a negative regulator. This is in contrast to *OsEF1Bγ*, which acts as a positive regulator of tolerance.

### *OsEF1Bγ* overexpressing and *OsYchF1* knockout mutant lines display increased detoxification capacity

To determine whether the enhanced stress tolerance of *OsEF1Bγ* overexpressing and *OsYchF1* knockout lines was attributable to improved detoxification capacity, we quantified levels of malondialdehyde (MDA), a hallmark of oxidative lipid damage. Abiotic stress induces oxidative stress by generating reactive oxygen species (ROS), which can damage lipids, proteins, and DNA. ROS attack polyunsaturated fatty acids *via* lipid peroxidation, yielding products such as MDA, 4‐hydroxy‐2‐nonenal, and F2‐isoprostanes (Tsikas, 2017). Therefore, MDA levels serve as a direct indicator of oxidative lipid damage and were used here to evaluate the extent of oxidative injury in the transgenic lines under stress.

Under treatments with 15% PEG6000 and 100 mM NaCl, the *OsEF1Bγ* OE#1 displayed noticeably lower MDA contents in both leaves and roots compared to those of WT plants (Figure 6A,B). Conversely, *OsYchF1* knockout mutant plants exhibit notably lower MDA levels in leaves relative to WT plants under the same stress conditions (Figure 6D). In parallel, we also performed histochemical staining to detect H_2_O_2_ accumulation, indicated by reddish‐brown precipitate. Following exposure to 15% PEG6000 and 100 mM NaCl, all three *OsYchF1* mutant lines demonstrated considerably fainter staining compared to the WT plants, indicating reduced H_2_O_2_ accumulation (Figure 6C).

**Figure 6:**
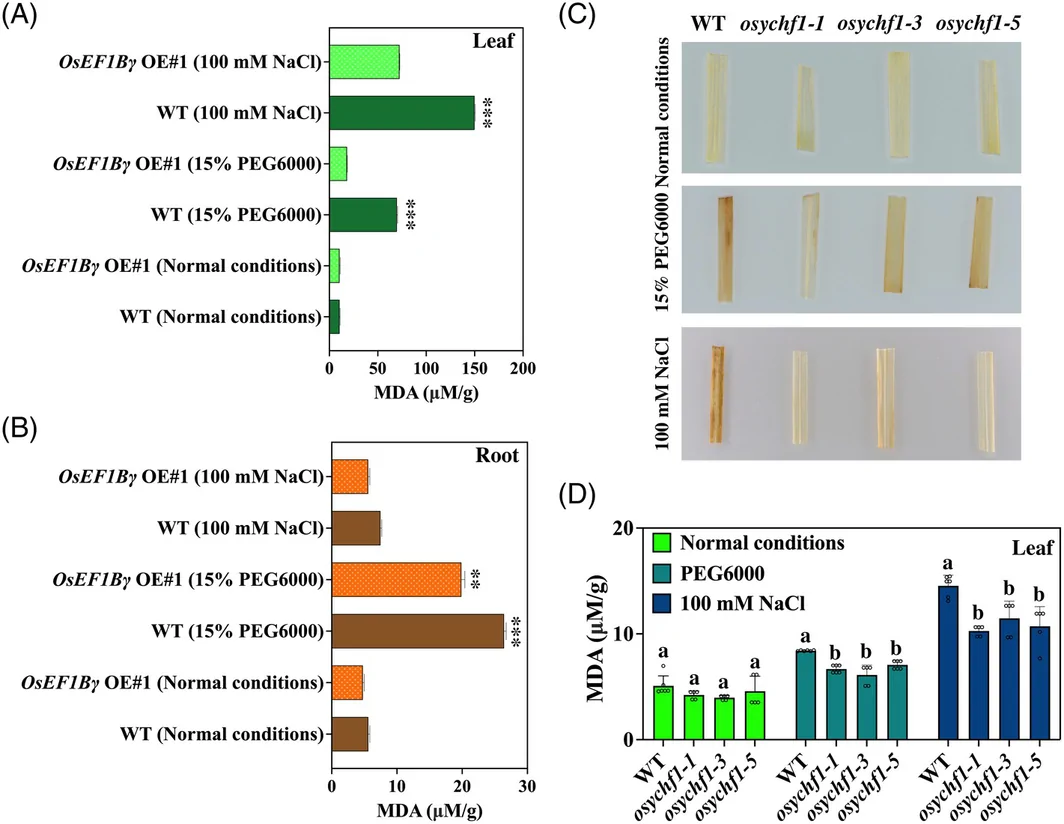
*OsEF1Bγ* overexpression lines and *OsYchF1* knockout mutants exhibit reduced oxidative damage under stressful conditions. (A, B) The malondialdehyde (MDA) content in the leaves (A) and roots (B) of the 8‐week‐old WT and *OsEF1Bγ* overexpression lines (OE#1) under normal conditions, 100 mM NaCl stress, and 15% PEG6000‐induced osmotic stress. *P < 0.05, **P < 0.01, ***P < 0.001 (Student's *t*‐test). (C) Histochemical detection of hydrogen peroxide (H_2_O_2_) using 3,3′‐diaminobenzidine (DAB) staining in the leaves of the 6‐week‐old WT and *Osychf1* mutants (*Osychf1‐1, Osychf1‐3, Osychf1‐5*) under normal conditions, 15% PEG6000‐induced osmotic stress, and 100 mM NaCl treatment. (D) MDA content in the leaves of the 6‐week‐old WT and *Osychf1* mutants (*Osychf1‐1, Osychf1‐3*, *Osychf1‐5*) under normal conditions, 15% PEG6000‐induced osmotic stress, and 100 mM NaCl treatment. Data are presented as mean ± SD (*n* ≥ 3). Different letters above the bars indicate statistically significant differences (*P* < 0.05) as determined by Tukey's HSD test. MDA content is expressed as μmol per gram of fresh weight (μM/g FW).

Interestingly, the MDA content in the roots of the *OsEF1Bγ* OE#1 was significantly lower than in WT roots under 100 mM NaCl stress, and this reduction was even more pronounced in the leaves (Figure 6A,B). This enhanced shoot protection likely reflects a delay in salt‐induced damage progression in aerial tissues, as well as tissue‐specific differences in oxidative stress management. Similar effects were also observed in the leaves of the *OsYchF1* mutants using 3,3′‐diaminobenzidine (DAB) staining (Figure 6C). Together, these results suggest that *OsYchF1* acts as a negative regulator, while overexpression of *OsEF1B*γ functions as a positive regulator in mediating cellular detoxification under stress conditions.

To determine whether enhanced tolerance depends directly on GST activity, we first measured GST activity in the *OsEF1Bγ* OE#1 and *osychf1‐1* knockout mutant lines. GST activity was noticeably higher in the *OsEF1Bγ* OE#1 and *osychf1‐1* lines than in the WT plants (Figure S6A,B). Secondly, we applied the well‐established GST inhibitor ethacrynic acid (EA) at a concentration of 200 μM to both the *OsEF1Bγ* OE#1 and the *osychf1‐1* lines under stressful conditions (15% PEG 6000 to simulate drought and a high salinity treatment of 100 mM) (Figure S7). As shown in Figure 1 and Figure S7, pharmacological inhibition of GST effectively eliminated the advantage of the *OsEF1Bγ*‐overexpressing and *OsYchF1*‐knockout mutant lines under stress conditions, indicating that their improved stress tolerance is indeed attributable to elevated GST activity.

To evaluate cellular antioxidant capacity, we quantified the enzymatic activities of catalase (CAT), peroxidase (POD), and superoxide dismutase (SOD) in both the *OsEF1Bγ* overexpression and *OsYchF1* knockout lines. The three enzymes constitute the frontline of the antioxidant defense network: SOD catalyzes the dismutation of superoxide radicals into hydrogen peroxide (H_2_O_2_), which is subsequently neutralized by the complementary actions of CAT and POD. Monitoring these activities therefore provides a comprehensive assessment of the ROS‐scavenging efficiency in the transgenic lines compared to the WT.

Under normal conditions, all *OsYchF1* knockout mutants (*osychf1‐1* and *osychf1‐5*) exhibited moderately higher basal CAT and SOD activities than WT plants. Notably, their POD activity was also significantly higher than that of WT plants (Figure S6C,E,G). Upon exposure to oxidative stress induced by 15% PEG6000 and 100 mM NaCl, the *OsYchF1* mutant lines exhibited significantly elevated antioxidant enzyme activities compared to WT plants (Figure S6C,E,G), suggesting a robust genotype‐dependent enhancement of the antioxidant response. Interestingly, *OsEF1Bγ* overexpression lines did not notably affect antioxidant activity relative to WT under stress (Figure S6B,D,F). Conversely, *OsEF1Bγ* OE#1 showed constitutively higher GST activity under normal conditions, unlike the *osychf1‐1* mutants (Figure S6A). Under stressful conditions, nevertheless, both *OsEF1Bγ* OE#1 and *osychf1‐1* mutant plants displayed significantly elevated GST activity compared to WT plants (Figure S6A).

Collectively, these findings indicate that OsEF1Bγ appears to play a more specialized role, primarily affecting GST activity, as it has negligible impact on major antioxidant enzymes (CAT, SOD, and POD). In addition to enhancing GST activity, knockout of *OsYchF1* augments both constitutive and stress‐induced antioxidant enzyme activities, which likely contributes to reduced oxidative damage. The enhanced ROS‐scavenging capacity of the *osychf1* mutants largely explains their reduced oxidative damage and improved growth performance under stressful conditions.

### Oxidative stress leads to OsYchF1 dimerization *in vitro* and decreased accumulation *in vivo*

First, treatment with 15% PEG6000 and 100 mM NaCl induces oxidative stress in rice. WT plants exhibit higher accumulation of hydrogen peroxide (H_2_O_2_) compared to *OsYchF1* knockout mutants under these osmotic and high salinity stress conditions (Figure 6C). Second, OsYchF1 contains five highly conserved surface‐exposed cysteine residues that may be susceptible to oxidative stress, thereby affecting its activity (Figure S8). For example, in bacterial YchF, the oxidation of a conserved cysteine residue (Cys^35^) within the G domain triggers intramolecular disulfide formation and homo‐dimerization. This structural shift downregulates its ATPase activity (Wenk et al., 2012). In contrast, Thioredoxin 1 (TrxA) interacts with the oxidized YchF to reduce the disulfide bond. This dissociates the dimer back into a monomeric state, thereby restoring and enhancing ATPase activity (Hannemann et al., 2016).

To recognize the underlying mechanism of OsYchF1 in response to the cellular oxidative stress changes caused by external stimuli, we analyzed OsYchF1's oligomerization state by a gel filtration assay in the presence or absence of dithiothreitol (DTT) *in vitro*. Recombinant (His)_6_‐OsYchF1 protein formed dimer or oligomer in the absence of DTT, but became monomeric in the presence of 1 mM DTT when subjected to a gel filtration assay (Figure 7A). As mentioned earlier, OsYchF1 can bind equally GTP or ATP in the NTP binding pocket (Figure 7B). We measured the GTPase activity of the monomeric and dimeric forms to test the sensitivity of OsYchF1 to oxidation. As a result, the OsYchF1 dimer displayed much lower GTPase activity than that of the OsYchF1 monomer (Figure 7C). Moreover, monomeric OsYchF1 is a functional protein sensitive to NTPase activation by the C2‐domain OsGAP1, whereas the dimeric OsYchF1 is refractory to OsGAP1, suggesting that dimeric oxidized forms of OsYchF1 are not functional proteins (Figure 7C). In agreement, the turbidity (indicating the formation of inactive aggregates) of OsYchF1 was also significantly greater than that of OsGAP1 (as a control) under 0.1 mM H_2_O_2_ treatment, suggesting that the OsYchF1 protein easily forms insoluble and inactive homo‐dimer or oligomer upon oxidative stress *in vitro* (Figure 7D).

**Figure 7:**
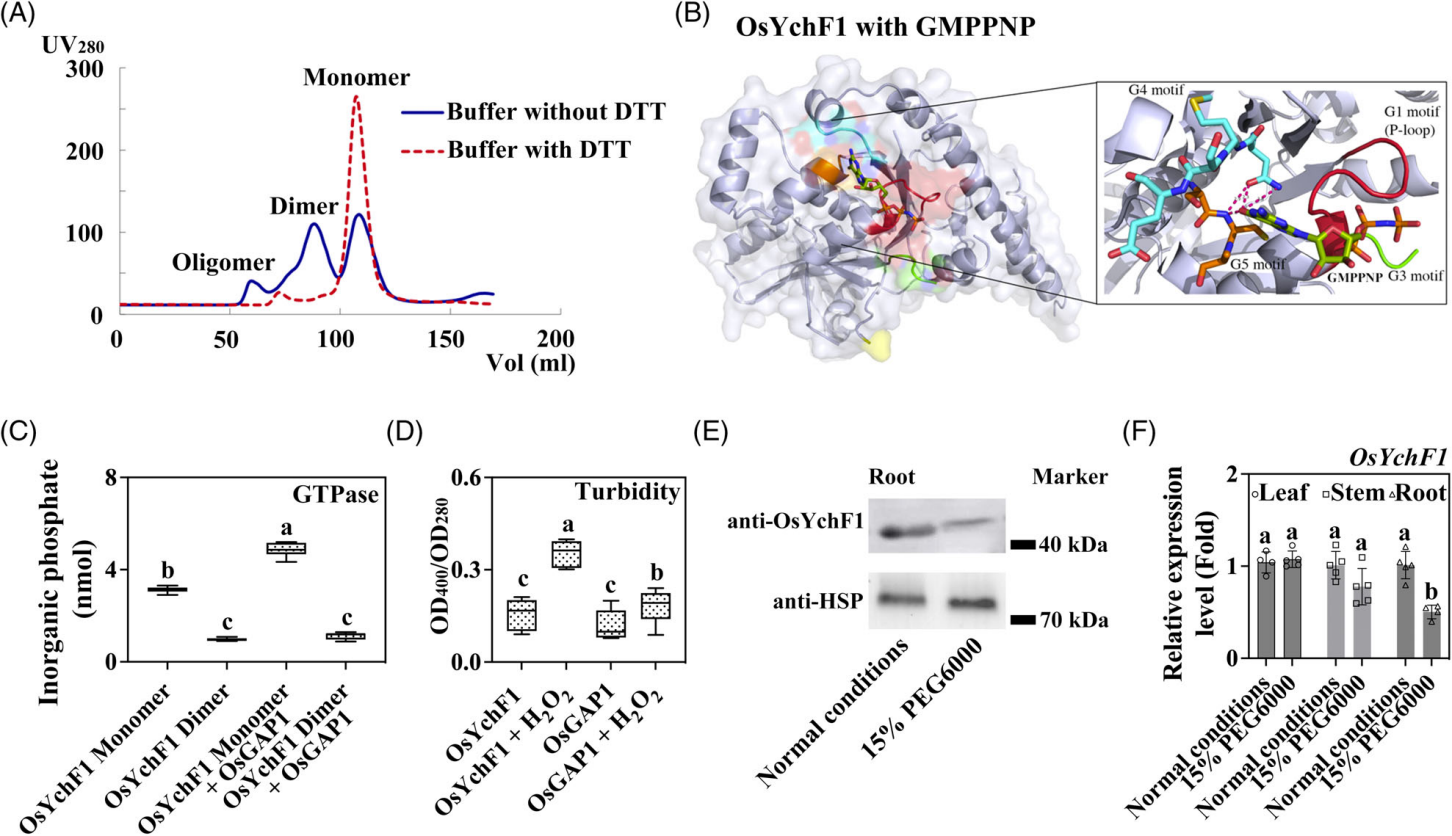
Oxidative stress induces OsYchF1 polymerization *in vitro* and reduces its accumulation *in vivo*. (A) Gel filtration profiles of OsYchF1 in the absence or presence of 1 mM DTT through monitoring OD_280_ (vertical axis). (B) Crystal structure of OsYchF1 in the complex with a non‐hydrolyzable GTP analog GMPPNP (PDB code: 5EE9). The G1‐G5 motifs were indicated as follows: G1 motif (pink), G2 motif (yellow), G3 motif (green), G4 motif (cyan), and G5 motif (brown). GMPPNP was shown as stick. (C) The OsYchF1 GTPase activity was measured based on the release of inorganic phosphate (Pi) using 200 μM GTP as the substrate. (D) The OsYchF1 turbidity (OD_400_/OD_280_) measurements in the absence or presence of 0.1 mM H_2_O_2_. Error bars mean the SD (*n* = 4); each experiment was repeated at least three times. Values with the same letters were not significantly different from one another (one‐way ANOVA, *P* < 0.05). (E) Immunoblot analysis of OsYchF1 protein abundance in WT roots under normal conditions and 15% PEG6000 treatment, using polyclonal antibodies raised against a peptide (amino acids 135–149: THIDDSVDPVRDLET) of OsYchF1and anti‐HSP82 (Heat Shock Protein 81–2) as a control. (F) The relative expression of *OsYchF1* in WT leaves, stems, and roots was investigated using qRT‐PCR under normal conditions and after treatment with 15% PEG6000. Error bars indicate the SD; *n* = 4, and each experiment was repeated at least three times. Values with the same letters are not significantly different from one another (one‐way ANOVA, *P* < 0.05).

Moreover, soluble protein extracts from the roots of WT seedlings grown under normal or osmotic stress (15% PEG6000) conditions (applied for 4 weeks following an initial 4‐week normal growth period) were separated by sodium dodecyl sulfate‐polyacrylamide gel electrophoresis (SDS‐PAGE). *In vivo* immunoblotting analysis revealed that OsYchF1 protein accumulated to higher levels in WT seedlings under normal conditions than in those subjected to 15% PEG6000 treatment (Figure 7E), indicating that PEG6000‐induced oxidative stress may reduce soluble OsYchF1 accumulation *in vivo*. In line with the immunoblotting analysis, qRT‐PCR revealed that *OsYchF1* transcript levels were significantly lower under 15% PEG6000 treatment compared to normal growth conditions in roots, although no significant differences were observed in leaves and stems (Figure 7F). Therefore, transcriptional downregulation likely contributes to the reduced protein level specifically in roots. However, we cannot exclude the possibility that translational inhibition and enhanced protein degradation at the post‐translational level are also involved, particularly given that transcript levels remain unchanged in the leaves and stems.

## DISCUSSION

The central finding of this study is the identification of a post‐translational regulatory mechanism that directly links the translation machinery to cellular detoxification. We demonstrate that the unconventional G protein OsYchF1 functions as a molecular gatekeeper for the GST activity of the elongation factor OsEF1Bγ. By binding to OsEF1Bγ, OsYchF1 impedes GSH access and inhibits its enzymatic function. This places OsYchF1 as a key negative regulator in a pathway where OsEF1Bγ acts as a positive effector of stress tolerance (Figure 8). Moreover, stress‐induced downregulation of OsYchF1 serves as a molecular trigger to activate the antioxidant defense system. Under non‐stress conditions, OsYchF1 maintains OsEF1Bγ in an inactive state through direct physical sequestration, preventing unnecessary metabolic flux. Upon stress perception, the reduction in OsYchF1 levels releases this molecular brake, enabling the rapid induction of OsEF1Bγ‐mediated GST activity to mitigate oxidative damage (Figure 8).

**Figure 8:**
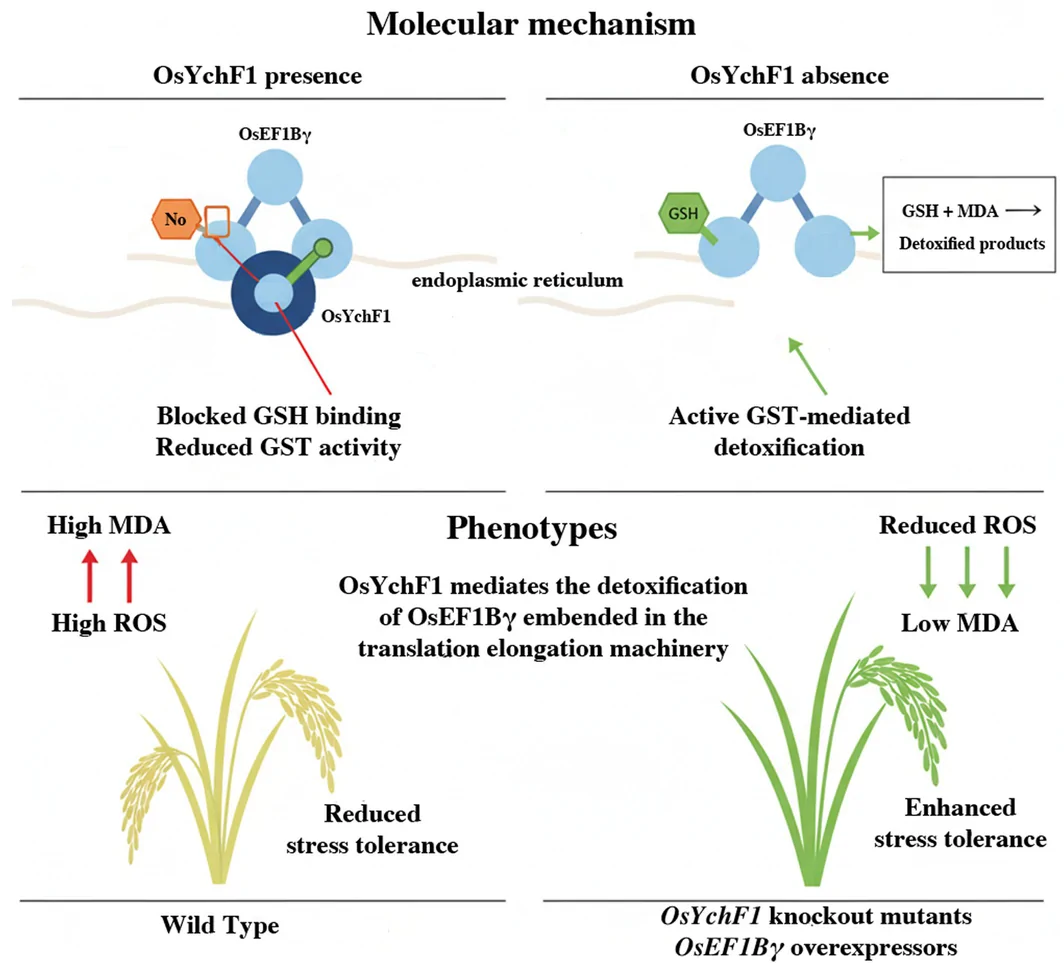
Proposed molecular mechanism of the OsYchF1‐mediated stress‐induced detoxification by regulating the accessibility of GSH to OsEF1Bγ. The diagram illustrates a proposed model in which OsYchF1 negatively regulates the glutathione S‐transferase (GST) activity of OsEF1Bγ at the endoplasmic reticulum. Molecular mechanism (Top): When OsYchF1 is present, it interacts with OsEF1Bγ and reduces GSH binding, leading to decreased GST activity and impaired detoxification. When OsYchF1 is absent, GSH binding to OsEF1Bγ is less inhibited, allowing GST‐mediated conjugation of GSH with MDA to form detoxified products. Corresponding phenotypes (Bottom): Wild‐type plants (OsYchF1 present) accumulate higher ROS and MDA levels and show reduced stress tolerance. In contrast, *OsYchF1*‐deficient plants or *OsEF1Bγ*‐overexpressing plants exhibit lower ROS and MDA levels and enhanced stress tolerance.

Our regulatory model aligns with and builds upon the long‐standing view that eEF1Bγ proteins, despite possessing a GST‐like fold, lack the essential catalytic amino acid residues required for classical GST activity, exhibiting negligible activity toward standard substrates (Dixon & Edwards, 2010; Oakley, 2011). Consequently, these proteins are generally considered to have evolved toward non‐catalytic, structural, or regulatory functions, including metabolite binding, cofactor interaction, and redox homeostasis. However, the rice OsEF1Bγ orthologue has been reported to exhibit low, yet measurable GST activity (Kobayashi et al., 2001; Lohmann et al., 2023). Nevertheless, our findings reveal that this activity is not constitutive, but is actively restrained by OsYchF1 under normal conditions. Thus, the traditional view of eEF1Bγ as primarily non‐catalytic remains a critical baseline, onto which stress‐induced activation, mediated by OsYchF1 downregulation, can be superimposed. Overall, OsEF1Bγ likely has a dual nature, functioning as a GST enzyme to enhance ER‐based detoxification and confer abiotic stress tolerance, while also serving non‐enzymatic ligand‐binding roles.

The YchF subfamily is composed of a group of highly conserved P‐loop NTPases capable of binding and hydrolyzing both GTP and ATP (Cheung et al., 2016; Koller‐Eichhorn et al., 2007). These enzymes play a role in vital cellular processes across diverse organisms, from bacteria to mammals, including oncogenesis, protein synthesis, and the stress response (Balasingam et al., 2020). Knockdown of *human Obg‐like ATPase 1* (*hOLA1*), a YchF orthologue present in humans, increases resistance to oxidizing agents without altering the mRNA levels of antioxidant enzyme genes, implying a non‐transcriptional role for YchF in the stress response (Zhang et al., 2009). Similarly, *E. coli* YchF functions independently of changes in the transcription or translation of H_2_O_2_‐scavenging enzymes after H_2_O_2_ exposure, further suggesting its involvement in post‐translational regulation (Hannemann et al., 2016). The overexpression of *E. coli YchF* leads to H_2_O_2_ hypersensitivity by inhibiting H_2_O_2_‐induced KatG activity (Wenk et al., 2012).

In plants, overexpressing *AtYchF1* (an Arabidopsis YchF) elevates ROS levels in transgenic BY‐2 cells (Cheung et al., 2013). Conversely, an *AtYchF1* knockdown mutant in Arabidopsis enhances tolerance to salinity‐induced oxidative stress by promoting antioxidant enzymatic activity (Cheung et al., 2013). Consistently, the current study found that the antioxidant enzymatic activities of CAT, POD, and SOD increased noticeably in rice *OsYchF1* knockout mutant lines (Figure S8). Intriguingly, the growth phenotype of the *OsYchF1* knockout mutants was almost the same as that of WT plants in normal conditions. Nevertheless, *OsYchF1* knockout mutants showed reduced MDA contents and enhanced antioxidant activities in the leaves under the osmotic stress induced by 15% PEG6000 treatment and under high salinity stress induced by 100 mM NaCl (Figure 6D). Compared with WT plants, *OsYchF1* knockout mutants demonstrated greater biomass under osmotic stress.

On the other hand, a growing body of evidence has demonstrated that eEF1Bγ acts as a structural scaffold for eEF1Bα, facilitating the binding of the complex to the ER and is crucial for the stress response in eukaryotes (Dever et al., 2018; Korostelev, 2022; Sanders et al., 1996). Under heat stress, McLoughlin et al. (2016) reported that small heat shock proteins (sHSPs) act together with HSP101 as molecular chaperones to protect eEF1Bγ and eEF1Bβ in cytosolic stress granules. This assists the resolubilization of eEF1B subunits during recovery from heat stress (McLoughlin et al., 2016). Furthermore, Lohmann et al. (2023) demonstrated that upon heat stress, the eEF1B subunits altered their localization pattern and accumulated in heat‐induced cytoplasmic foci with properties similar to stress granules (SGs).

An *Aspergillus fumigatus eEF1Bγ* loss‐of‐function mutant showed much more sensitivity to the oxidants (H_2_O_2_, diamide, and 4,40‐dipyridyl disulfide (DPS)) than the WT (O'Keeffe et al., 2013). Furthermore, research on *Phanerochaete chrysosporium* eEF1Bγ (PcEF1Bγ) revealed that it physically interacts with other GSTs belonging to the Ure2p class in the presence of oxidized glutathione (GSSG) (Bchini et al., 2021). This interaction suggests a potential mechanism through which cellular redox balance may be coupled to stress response regulation by modulating PcEF1Bγ activity (Bchini et al., 2021). In addition to GST activity, GSTs may exhibit peroxidase, reductase, dechlorinase, and isomerase activities (Labrou et al., 2015; Nianiou‐Obeidat et al., 2017; Sylvestre‐Gonon et al., 2019). It is therefore possible that OsEF1Bγ participates in metabolite binding, cofactor interaction, or redox homeostasis.

In plants, evidence suggests that eEF1Bγ plays a critical role in the development and stress adaptation (Lohmann et al., 2023; McLoughlin et al., 2016). Homozygous double mutants (*eef1bγ1–2 eef1bγ2–2*) in Arabidopsis exhibit growth retardation, which links this factor to developmental regulation (Lohmann et al., 2023). In contrast to this loss‐of‐function phenotype, our research in rice suggests that the overexpression of *OsEF1Bγ* promotes greater biomass accumulation in both the shoot and the root, suggesting a gain‐of‐function phenotype.

While the GST activity of eEF1Bγ is documented in fungi (Allocati et al., 2014; Carberry et al., 2006), its regulatory mechanism remains largely unknown. Building upon the established role of eEF1Bγ in stress adaptation, our study reveals a novel regulatory mechanism for its function. We identified that in rice, OsYchF1 interacts with OsEF1Bγ in the endoplasmic reticulum and functions as a negative regulator by impeding the binding of GSH, thereby inhibiting its GST activity. This not only explains the enhanced antioxidant capacity observed in *OsYchF1* knockout mutants but also establishes a direct biochemical link between the YchF family and the regulation of primary metabolism (translation) during stress.

Moreover, previous studies have shown that OsYchF1 binds to its activator OsGAP1 *via* the G and TGS domains, though this interaction is sterically hindered by its helical domain (Huang et al., 2025). Here, we found that all OsYchF1 domains are implicated in interacting with the two bi‐terminal domains of OsEF1Bγ *in vivo*, and that this interaction inhibits the GST activity of OsEF1Bγ *in vitro*. These results suggest that OsYchF1 docks into OsEF1Bγ in a plug‐like manner and suppresses its enzymatic activity.

While our biochemical and phenotypic data provide strong evidence supporting the core model, we must transparently acknowledge a key limitation of the current study. Without formal genetic epistasis, we cannot definitively exclude the possibility of additional, independent minor contributions from the other components within this regulatory network. The observed phenotypes, while consistent with our proposed mechanism, could partially reflect parallel pathways acting in concert. To address this, we have prioritized the construction and phenotypic characterization of the *osychf1 osef1bγ* double mutants. This genetic approach, followed by targeted complementation analyses, will be essential to systematically deconstruct the functional hierarchy. These future experiments are critical for distinguishing between a linear dependency and a more complex, additive relationship between these two factors, thereby providing a definitive genetic framework to solidify our mechanistic model.

Furthermore, as shown in Table S1, there are approximately 30 distinct translation‐related protein groups. The identified proteins span multiple functional categories of the translational machinery, including initiation factors (e.g., eukaryotic initiation factor 4A isoforms and eIF5), elongation factors (e.g., eEF1A, eEF1Bγ, eEF2, and chloroplastic EF‐Tu), and a wide array of ribosomal proteins (including 40S subunits and 60S subunits). We believe that translational inhibition should be also responsible for the decreased accumulation of OsYchF1.

Members of the YchF family have been reported to be involved in protein degradation (Gradia et al., 2009; Guerrero et al., 2006). Further research into YchF‐mediated protein degradation could reveal a new mechanistic link between OsYchF1 and either the ubiquitin–proteasome system (UPS) or autophagy‐mediated turnover under osmotic stress. Indeed, our IP‐MS screening has already identified several potential OsYchF1 interacting partners directly involved in ubiquitination and proteasomal degradation pathways, as shown in Table S1 (e.g., ubiquitin fused to ribosomal protein L40). Consistent with this, numerous studies have demonstrated that YchF family proteins in other species act as scaffolding hubs that recruit E3 ubiquitin ligases or deubiquitinases to modulate the stability of stress‐responsive targets (Gradia et al., 2009; Guerrero et al., 2006).

Overall, our data support a model in which, under non‐stress conditions, OsYchF1 binds to OsEF1Bγ, maintaining OsEF1Bγ's detoxification activity in a “low‐flux” state (Figure 8, Closed). This may be crucial for preserving cellular redox balance for normal growth. Upon stress perception, the inhibition is relieved through oxidative stress‐caused downregulation of OsYchF1, which disrupts the OsYchF1‐OsEF1Bγ interaction and allows OsEF1Bγ to bind GSH and detoxify lipid peroxidation products, such as MDA (Figure 8, Open). This gating mechanism provides a rapid, post‐translational means to enhance cellular detoxification capacity without requiring de novo protein synthesis. Future work should aim to identify the upstream signals and potential post‐translational modifications (e.g., ubiquitination, phosphorylation) that govern the dynamic assembly and disassembly of this regulatory complex. Furthermore, given the structural tractability of the GSH‐binding pocket identified in our model (Figure 4A), it represents a promising biotechnological target for engineering climate‐resilient crops that can better balance growth and stress adaptation. More specifically, we propose a feasible gene‐editing strategy: targeted editing of the *OsEF1Bγ* coding region *via* CRISPR/Cas9 to generate alleles with moderately elevated GST activity and enhanced stress tolerance, enabling the breeding of stress‐resilient rice varieties without a growth penalty.

## METHODS

### Plant materials and growth conditions

Wild‐type (WT) *O. sativa* L. ssp. japonica (cv. Nipponbare), transgenic rice plants overexpressing *OsEF1Bγ*, and *OsYchF1* knockout mutants generated by CRISPR‐Cas9‐based gene editing were cultivated in a paddy field in Fuzhou, Fujian, China under local conditions or in a growth chamber with 14‐h light/10‐h darkness cycles at a constant 29°C, and the light intensity was 200 μmol photons m‐2 sec‐1. 4‐week‐old seedlings' leaf, stem, or root samples were harvested and rapidly frozen in liquid nitrogen or stored at −80°C for further expression analysis by quantitative real‐time PCR (qRT‐PCR; *Actin1* used as an internal control gene) or other experiments. Whole plants were harvested. Shoots and roots were dried at 60°C for 1 week before measuring their dry weight.

### Generation of transgenic rice plants overexpressing *OsEF1Bγ*

The full‐length coding sequence (CDS) of *OsEF1Bγ* was amplified by PCR using PrimeSTAR Max DNA Polymerase (Cat. No. R045A, Takara Bio USA, Inc.) with a denaturation step at 95°C for 5 min, followed by 35 cycles of denaturation at 94°C for 30 s, annealing at 55°C for 5–10 s, and extension at 72°C for 1 min, with a final extension step at 72°C for 5 min. The digested DNA products of full‐length *OsEF1Bγ* were then cloned into the plant expression vector pCAMBIA2301 (*EcoR*I and *Sac*I), which is driven by the cauliflower mosaic virus (CaMV) 35S promoter (35S::OsYchF1‐FLAG). All verified constructs were transformed into the rice cultivar Nipponbare using the GV3101 Agrobacterium strain by EDGENE Biotechnology (Wuhan, China).

### Generation of *OsYchF1* ‐knockout mutants by Crispr‐Cas9‐based gene‐editing approach

EDGENE Biotechnology (Wuhan, China) produced *OsYchF1* knockout mutants in the rice cultivar Nipponbare using the CRISPR‐Cas9‐based gene‐editing approach.

### Immunoprecipitation‐mass spectrometry (IP‐MS) analyses

Proteins were extracted from 4‐week‐old FLAG‐tagged OsYchF1 rice seedlings grown in liquid medium. Pulverized leaves were homogenized in ice‐cold extraction buffer (100 mM Tris–HCl pH 7.5, 200 mM NaCl, 2 mM EDTA, 20% glycerol, 0.4% Triton X‐100, and 2× protease inhibitors) and centrifuged at 13000 ***g*** for 30 min at 4 C. The supernatant was immunoprecipitated with FLAG‐Trap agarose beads (Sigma‐Aldrich) at 4 C for 3 h. Beads were washed five times with 50 mM Tris‐Cl (pH 7.5) containing 0.1% Triton X‐100 and eluted with protein loading buffer. The Coomassie‐stained OsYchF1‐FLAG band was excised and analyzed by LC–MS/MS using a Q Exactive mass spectrometer coupled with an Easy nLC system (Thermo Fisher Scientific) at Biogreen Biotechnology (Hangzhou) Co., Ltd.

### Firefly luciferase complementation imaging (LCI) assay

To generate constructs for LCI assay, the coding sequences of the full‐length *OsEF1Bγ* and its truncated derivatives including N‐terminal domain (amino acid (aa) 1–208) and C‐terminal domain (aa 240–416), as well as the full‐length *OsYchF1* and its truncated derivatives including N‐terminal core G domain (aa 1–128), inserted helical domain (aa 141–199), C‐terminal TGS domain (aa 303–394) and No TGS domain containing both G and helical domains (aa 1–302) were subcloned into the pCAMBIA‐1300NLuc (*Kpn*I and *Sal*I), pCAMBIA‐1300CLuc (*Kpn*I and *BamH*I) or pCAMBIA‐1300CCLuc (*Kpn*I and *Sal*I) using T4 DNA ligase, respectively. These constructs were co‐transformed into the Agrobacterium strain GV3101 and infiltrated into the fully expanded leaves of 5–6 week‐old *N. benthamiana* plants. Two days later, the *N. benthamiana* leaves were treated with 1 mM luciferin (Cat. No. E160A; Promega), maintained in darkness for 5 min and photographed using a low‐light cooled 644 charge‐coupled device camera (Ikon‐L936, Andor Technology). Luciferase signals were detected as described by Miao et al. (2019). Primers are listed in Table S2.

### Bimolecular fluorescence complementation (BiFC) assay

For the BiFC assay, the coding sequences of the full‐length *OsEF1Bγ* and its truncated derivatives, as well as the full‐length *OsYchF1* and its truncated derivatives, were subcloned into the pCAMBIA1300‐N1‐GFPN and pCAMBIA1300‐N1‐GFPC vectors. This yielded the following constructs: OsYchF1‐nGFP, OsYchF1 G domain‐nGFP, OsYchF1 helical domain‐nGFP, OsYchF1 TGS domain‐nGFP, and OsYchF1 No TGS domain‐nGFP, as well as OsEF1Bγ‐cGFP, OsEF1Bγ‐N‐cGFP, and OsEF1Bγ‐C‐cGFP. The BiFC assay was performed as previously described (Miao et al., 2018; Miao et al., 2021; Yuan et al., 2018). The GFP fluorescence images were obtained by using a Leica SP8 confocal laser scanning microscope (Leica, Heidelberg, Germany). The primers used are listed in Table S2.

### Structure prediction and molecular docking

The dimeric OsEF1Bγ structure prediction was performed using AlphaFold3 (https://alphafoldserver.com/welcome). The predicted Protein Data Bank (PDB) files produced by AlphaFold3 were then imported into PyMOL for visualization. The rank‐1 file with the highest pLDDT score was selected for further analysis. The protein structures were prepared by adding hydrogen atoms, repairing side chains, and removing water molecules, followed by energy minimization. The glutathione (GSH)‐binding pocket was identified using AutoDock Vina 1.1.2 (https://vina.scripps.edu), and the GSH ligand was docked into this site. Twenty binding poses were generated and exported for further analysis (Eberhardt et al., 2021; Trott & Olson, 2010).

### Molecular dynamics simulations

The molecular dynamics (MD) simulation was performed using Amber24 software with a simulation duration of 100 ns. Prior to the MD simulations, the GSH parameters were set through GAFF, and the OsEF1Bγ parameters were depicted using ff19SB from the Amber software. The complex was immersed in a TIP3P water box, leaving a 10 Å buffer space between the protein and the box boundaries. An appropriate number of Na^+^ counterions was then added to reduce the total number of static charges to zero. Energy minimization of the eight complexes was implemented by the steepest descent and conjugate gradient methods. The eight complexes were slowly heated from 0 to 300 K in the NVT (number of particles, volume, and temperature) ensemble, followed by unstrained equilibration at 300 K. To guarantee the uncorrelation of conformations, coordinate trajectories were saved every 2 ps. Finally, a 100 ns MD simulation was performed at a constant temperature of 300 K and pressure of 105 Pa using the PMEMD module. The binding free energy was then calculated based on the Molecular Mechanics Poisson‐Boltzmann Surface Area method. After the MD simulation, the structures were visualized using PyMOL software.

### Protein expression and purification

The digested fragments of the full‐length *OsEF1Bγ* and *OsYchF1* coding sequences (restricted with *Age*I and *EcoR*I or *Age*I and *Sac*I, respectively) were inserted into the minipRSETA‐hisSUMO expression vector using T4 DNA ligase. The digested *OsGAP1* fragment was inserted into the pGEX‐4 T‐1 expression vector (restricted with *BamH*I and *Xho*I). These plasmids were then used to transform *Escherichia coli* (*E. coli*) DH5α. Transformants containing the *OsEF1Bγ*, *OsYchF1*, and *OsGAP1* constructs were screened by colony PCR using the T7/T7 terminator or pGEX5’/pGEX3’ primer pairs. All constructs were verified by Sanger DNA sequencing across the cloning sites to ensure the production of in‐frame N‐terminal 6x polyhistidine (His_6_) tagged or glutathione S‐transferase (GST) tagged fusion proteins. All primers used for plasmid construction are listed in Table S2. *E. coli* BL21 (DE3) pLysS competent cells were transformed with the expression vectors containing the full‐length *OsEF1Bγ*, *OsYchF1*, and *OsGAP1* coding sequences. Protein expression and purification were performed as previously reported (Miao et al., 2019).

### Glutathione S‐transferase (GST) activity assay

GST activity was measured using a spectrophotometric method (Kobayashi et al., 2001). The reaction mixture was made up of a total volume of 200 μL and contained 50 mM potassium phosphate buffer solution (pH 7.5), 0.5 mM reduced form glutathione (GSH), 0.5 mM 1‐chloro‐2,4‐dinitrobenzene (CDNB), and 0.1 μM of purified OsEF1Bγ, in the presence of either 0.1 μM OsYchF1 or, for the control, 0.1 μM of bovine serum albumin (BSA). Absorbance was recorded continuously at 340 nm over a 15‐min period in a UV/visible spectrophotometer at 25°C. GST activity in rice leaves was measured spectrophotometrically using the 0.5 mM CDNB. The increase in A_340_ was measured in a 3 mL reaction mixture over 3 min (Mauch & Dudlar, 1993). These rate measurements were then converted to μmol of CDNB conjugated per min per mg of protein using an extinction coefficient of 9.6 mmol L^−1^ cm^−1^ (Lares et al., 2023).

### Isothermal titration calorimetry (ITC) measurements

The interactions of purified OsEF1Bγ with glutathione (GSH), either in the presence of OsYchF1 or as a control with bovine serum albumin (BSA), were analyzed using a MicroCal ITC200 system (Malvern Panalytical) as previously described (Miao et al., 2019). Prior to ITC measurements, all proteins were dialyzed against a dialysis buffer containing: 20 mM Tris–HCl pH 7.5, 200 mM NaCl, and 1 mM DTT at 4°C overnight. GSH (500 μM) in the syringe was titrated into a chamber containing purified OsEF1Bγ (60 μM), either in the presence of OsYchF1 (60 μM) or BSA (60 μM). The ITC experiments were repeated twice with similar results at 25°C.

### Gel filtration assay

OsYchF1 proteins purified as described above were concentrated to 2 mg/mL and subjected to a preparative grade HiLoad 26/60 Superdex 200 gel filtration column (GE Healthcare, Uppsala, Sweden, 17‐1043‐01) equilibrated with 20 mM Tris, 200 mM NaCl, pH 7.5 buffer in the absence or presence of 1 mM DTT.

### MDA measurements

A total of 0.2 g of fresh rice leaf and root tissue was ground in liquid nitrogen and homogenized in 1.6 mL of 0.1% (w/v) trichloroacetic acid (TCA). The homogenate was centrifuged at 10000 ***g*** for 15 min at 4°C, and 1.5 mL of the resulting supernatant was reacted with 1.5 mL of 0.67% (w/v) thiobarbituric acid (in 20% TCA). This mixture was heated in a boiling water bath for 30 min, cooled to room temperature, and centrifuged briefly before measuring absorbance at 450, 532, and 600 nm. The MDA concentration (C, μmol/L) was calculated using the formula (C = 6.45 × (OD_532_ – OD_600_) – 0.56 × OD_450_) and the final content was expressed as μmol/g fresh weight (FW) using the equation (C × V)/W, where V represents the extraction volume (1.6 mL) and W represents the sample fresh weight (0.2 g) (Tsikas, 2017).

### Catalase (CAT) activity assays

To determine catalase (CAT) activity, the ultraviolet absorption method, as described by Aebi (1984), was used to measure the rate of H_2_O_2_ decomposition. A reaction mixture was prepared by adding 0.3092 mL of 30% H_2_O_2_ to 200 mL of 0.15 mol/L phosphate buffer solution (pH 7.0). 3 mL of this mixture was then mixed with 0.1 mL of the enzyme extract to initiate the reaction. Decreases in absorbance at 240 nm were monitored at 10‐s intervals over a 1‐min period. A control blank using phosphate buffer instead of the enzyme extract was used for zero adjustment. One unit of CAT activity (U) was defined as a 0.01 unit decrease in absorbance per minute. Final activity was expressed as U/g fresh weight (FW) using the formula: CAT activity (U/g FW) = (ΔA240 × Vt)/(W × Vs × 0.01 × t). In this equation, ΔA240 represents the change in absorbance at 240 nm, Vt is the total extraction buffer volume (1.6 mL), W is the sample fresh weight (0.2 g), Vs is the enzyme extract volume used in the assay (0.1 mL), and t is the reaction time (1 min).

### Peroxidase (POD) activity assay

Peroxidase (POD) activity was measured using the guaiacol method described by Chance and Maehly (1955). The reaction solution was prepared by dissolving 42 μL of guaiacol in 75 mL of 100 mM phosphate buffer solution (pH 7.0) at a gentle heat. Once cooled, 28.5 μL of 30% H_2_O_2_ was added. For the assay, 10 μL of the enzyme extract was mixed with 300 μL of the reaction solution in a microplate well. A mixture of 300 μL of the reaction solution and 10 μL of distilled water was used as a blank. The increase in absorbance at 470 nm, resulting from the oxidation of guaiacol, was monitored every 30 s for 4 min. One unit of POD activity (U) was defined as an increase in absorbance of 0.01 per minute. Activity was calculated using the formula POD activity (U/g FW) = (ΔA470/t), where ΔA470 is the change in absorbance over the reaction time, t is the total reaction time (4 min).

### Superoxide dismutase (SOD) measurements

Superoxide dismutase (SOD) activity was determined using the nitroblue tetrazolium (NBT) photoreduction method, as described by Beauchamp and Fridovich (1971). A reaction master mix was prepared by combining 105 mL of 50 mM phosphate buffer solution (pH 7.8) with 15 mL each of 130 mM methionine, 750 μM NBT, and 100 μM EDTA‐Na_2_, plus 12 mL of distilled water. For each assay, 0.1 mL of enzyme extract was added to 5.4 mL of the reaction mixture, followed by the addition of 500 μL of 20 μM riboflavin. Control tubes were substituted with distilled water for the extract. One control tube was kept in total darkness to serve as a blank, while the others were incubated under constant light for 20 min to initiate the photochemical reaction. Following incubation, absorbance was measured at 560 nm. One unit of SOD activity (U) was defined as the amount of enzyme that achieved 50% inhibition of NBT photoreduction. Activity was quantified using the formula: SOD activity (U/g FW) = [(A_control – A_sample) × Vt]/(0.5 × A_control × W × Vs), whereA_control and A_sample represent the absorbance of the irradiated control and sample respectively, Vt is the total extraction volume (1.6 mL), W is the fresh weight (0.2 g), and Vs is the enzyme extract volume used in the assay (0.1 mL).

### DAB staining

The accumulation of H_2_O_2_ in WT and *Osychf1* knockout mutant leaf tissues was visualized histochemically using 3,3′‐diaminobenzidine (DAB) as a substrate, following a modified protocol (Thordal‐Christensen et al., 1997). Freshly harvested rice leaves were first immersed in a DAB staining solution (1 mg/mL DAB, pH 3.8). Vacuum infiltration of the staining solution into the leaf tissue was then facilitated by placing the samples in a vacuum desiccator for 10 mins, followed by incubation in the dark at room temperature for approximately 8 h. After this period, the DAB solution was removed, and the leaf samples were transferred to 95% ethanol. The samples were then boiled in a water bath until the chlorophyll was completely removed, resulting in de‐stained leaves. The presence of H_2_O_2_ was indicated by the formation of reddish‐brown precipitates at the sites of its accumulation within the leaf tissue. The stained leaves were then rinsed and mounted in distilled water for observation.

### P‐loop NTPase activity assay

P‐loop NTPase activity of purified and separated (His)_6_‐OsYchF1 monomer and dimer in the absence or presence of GST‐OsGAP1 was measured by determining the release of inorganic phosphate (Pi) during GTP hydrolysis using the EnzChek™ Phosphate Assay Kit (E6646; Molecular Probes, Carlsbad, CA, USA) according to a previous method (Cheung et al., 2008).

### Western blots

For protein extraction, the roots of 4‐week‐old WT Nipponbare rice plants (100 mg) were collected and lysed in 600 μL extraction buffer (50 mM Tris–HCl, pH 7.5, 150 mM NaCl, 1 mM EDTA, 0.1% SDS, 1% Triton X‐100, and 1 mM dithiothreitol) with 1% protease inhibitor cocktail (04693116001, Roche). A Bradford protein assay was conducted to monitor the total protein separated on an SDS‐PAGE gel before electrophoretic transfer to a Hybond‐C membrane (Amersham Pharmacia Biotech USA, Inc.) using a Trans‐Blot Semi‐Dry Transfer cell (Bio‐Rad Laboratories USA, Inc.). 20 μg total protein was loaded per well in 10% SDS‐PAGE gel. OsYchF1‐specific polyclonal antibodies generated in rabbits using a synthetic peptide (THIDDSYDPVRDLET) corresponding to amino acid (aa) 135–149 of OsYchF1 were used to determine the OsYchF1 cross‐reacting bands in western blot analyses. The GE Amersham Imager 600 was used for observing cross‐reacting bands in Western blots.

### Statistical analysis

The data were analyzed using the Statistical Package for the Social Sciences (SPSS). Student's *t*‐test or one‐way analysis of variance (ANOVA) with Tukey's post hoc test was used to analyze the mean difference.

## Author Contributions

RM planned and designed the research; LG, DC, RL, YW, YL, YM, LY, QL and RM collected the data; YM, GH and RM analyzed the data; RM drafted the manuscript. All authors have given approval to the final version of the manuscript.

## Conflict of Interest Statement

The authors have no relevant financial or non‐financial interests to disclose.

## Supporting information


**Figure S1.** The intact OsEF1Bγ interacts with all domains of OsYchF1. (A, B) Bimolecular fluorescence complementation (BiFC) assays demonstrate the *in vivo* interaction between OsEF1Bγ‐nGFP and truncated versions of OsYchF1, including OsYchF1 G domain‐cGFP, OsYchF1 Helical domain‐cGFP and OsYchF1 TGS domain‐cGFP in *Nicotiana benthamiana* leaf epidermal cells (A) and rice leaf protoplasts (B). Scale bars = 10 μm. Cells were imaged using confocal microscopy.
**Figure S2.** Structure‐based alignment of OsEF1Bγ using software Jalview version 1.6. This shows the structure‐based alignment of OsEF1Bγ (NP_001389794.1), OsEF1Bγ‐1 (BAA34206.1), and OsEF1Bγ‐3 (BAD61828.1), *Arabidopsis thaliana* EF1Bγ (NP_563848.1, NP_176084.1) and, *Saccharomyces cerevisiae* EF1Bγ (NP_015277.1, NP_012842.1). Identical amino acid residues are marked in dark blue and homologous residues in light blue. Gaps are marked as dashes. α‐helices are indicated by red lines and β‐strands by green lines. The predicted binding residues (Asn‐11 and Lys‐12) are marked with asterisks.
**Figure S3.** Molecular phylogenetic analysis of OsEF1Bγ and its homologs. OsEF1Bγ, OsEF1Bγ‐1, OsEF1Bγ‐3, AtEF1Bγ, and ScEF1Bγ were analyzed by the Maximum Likelihood method. The protein accession numbers are as follow: OsEF1Bγ (NP_001389794.1), OsEF1Bγ‐1 (BAA34206.1.1), OsEF1Bγ‐3 (BAD61828.1), AtEF1Bγ (NP_563848.1, NP_001031202.1), ScEF1Bγ (NP_015277.1, NP_012842.1). Bootstrap values are recorded at the nodes.
**Figure S4.** Sequence analysis of CRISPR‐Cas9‐mediated genome editing of *OsYchF1* in rice. (A) The homozygous mutant *osychf1‐1* carries a single‐nucleotide insertion of an “A” relative to the reference sequence, which is present in both Allele1 and Allele2. (B) The *osychf1‐3* mutant carries a homozygous deletion mutation. Both alleles show a nucleotide deletion “GTCG” relative to the reference sequence. (C) The homozygous mutant *osychf1‐5* carries a single‐nucleotide insertion of an “A” relative to the reference sequence, which is present in both Allele1 and Allele2. For each mutant, the anchor and degenerate sequences used for genotyping are indicated above the alignments. Sequence alignments compare the mutant alleles with the reference sequence; dashes denote gaps.
**Figure S5.** SDS‐polyacrylamide gel electrophoresis analysis of the purified proteins. Eluted fractions are shown in lanes 1, 2, and 3 for OsEF1Bγ, OsEF1Bγ (N11A, K12A), and OsYchF1, respectively. The predicted molecular masses of the His‐SUMO‐tagged OsEF1Bγ proteins (both wild‐type and the N11A, K12A mutant) and OsYchF1 are 65.4 kDa and 44.3 kDa, respectively. M, protein marker.
**Figure S6.** GST and antioxidant enzyme activity in the leaves of the WT, *OsEF1Bγ* overexpression and *OsYchF1* knockout mutant plants under normal and stressful conditions. (A) GST activity in the leaves of WT, plants overexpressing *OsEF1Bγ* (*OsEF1Bγ* OE#1) and *OsYchF1* knockout mutants (*osychf1‐1* and *osychf1‐5*) plants under normal conditions and under osmotic stress induced by 15% PEG6000 or salt stress induced by 100 mM NaCl. (B) Catalase (CAT) activity in the leaves of WT and *OsEF1Bγ* overexpression (*OsEF1Bγ* OE#1 and 3) under normal conditions, and under 15% PEG6000‐induced osmotic stress or 100 mM NaCl salt stress. (C) CAT activity in the leaves of WT and *OsYchF1* knockout mutants (*osychf1‐1, osychf1‐5*) under normal conditions, and under 15% PEG6000‐induced osmotic stress or 100 mM NaCl salt stress. (D) Peroxidase (POD) activity in the leaves of WT and *OsEF1Bγ* overexpression (*OsEF1Bγ* OE#1 and 5) under normal conditions, and under 15% PEG6000‐induced osmotic stress or 100 mM NaCl salt stress. (E) POD activity in the leaves of WT and *OsYchF1* knockout mutants (*osychf1‐1* and *osychf1‐5*) under normal conditions, and under 15% PEG6000‐induced osmotic stress or 100 mM NaCl salt stress. (F) Superoxide dismutase (SOD) activity in the leaves of WT and *OsEF1Bγ* overexpression (*OsEF1Bγ* OE#1 and 5) under normal conditions, and under 15% PEG6000‐induced osmotic stress or 100 mM NaCl salt stress. (G) SOD activity in the leaves of WT and *OsYchF1* knockout mutants (*osychf1‐1* and *osychf1‐5*) under normal conditions, and under 15% PEG6000‐induced osmotic stress or 100 mM NaCl salt stress. Error bars indicate ± SD (*n* = 3 in three independent experiments). Values with the same letters are not significantly different from one another (one‐way ANOVA, *P* < 0.05).
**Figure S7.** The GST inhibitor abolished the stress tolerance conferred by the *OsEF1Bγ* overexpression and *OsYchF1* knockout under stressful conditions. (A) Representative phenotypes of 8‐week‐old WT Nipponbare (Nip), a transgenic line overexpressing *OsEF1Bγ* (*OsEF1Bγ* OE#1) and a CRISPR‐Cas9‐based gene‐editing transgenic rice line (*osychf1‐1*) with Nip as the background are shown under normal conditions, after treatment with 15% PEG6000 to induce osmotic stress and after treatment with 100 mM NaCl. (B) The plant length of 8‐week‐old WT, *OsEF1Bγ* OE#1 and *osychf1‐1* plants under normal conditions. Plant length of 8‐week‐old WT, *OsEF1Bγ* OE#1 and *Osychf1‐1* plants with 200 μM ethacrynic acid (EA) is shown in (C) when subjected to osmotic stress caused by 15% PEG6000, and in (D) after 100 mM NaCl treatment. (E) Shoot and root dry weight of 8‐week‐old WT, *OsEF1Bγ* OE#1 and *Osychf1‐1* plants under normal conditions. Shoot and root dry weight of 8‐week‐old WT, *OsEF1Bγ* OE#1 and *Osychf1‐1* plants with 200 μM EA upon osmotic stress caused by 15% PEG6000 (F), and after 100 mM NaCl treatment (G). Scale bars = 10 cm. Error bars indicate the SD; *n* ≥ 5, and each experiment was repeated at least three times. Different letters above the bars indicate statistically significant differences (*P* < 0.05) as determined by the Tukey's HSD test.
**Figure S8.** Structure‐based alignment of OsYchF1 with its plant orthologs using Jalview software (version 1.6). gi/138637314 is OsYchF1 in rice, gi/242080845 is *Sorghum bicolor* YchF, gi/225444063 is *Vitis vinifera* YchF, gi/255564375 is *Ricinus communis* YchF, gi/15221444 is *Arabidopsis thaliana* YchF, gi/116789076 is *Picea sitchensis* YchF, gi/168048097 is *Physcomitrella patens* YchF. α‐helices (red lines) and β‐strands (green lines) are indicated below the sequences. Evolutionarily conserved cysteine residues are marked with red asterisks.
**Table S1.** OsYchF1 LC–MS/MS results (translation‐related) in rice.
**Table S2.** Primers used in the study.

## Data Availability

Correspondence and requests for materials should be addressed to Dr. Rui Miao.
