# Supplementary material for: OsYchF1 suppresses OsEF1Bγ‐mediated ER detoxification to co‐involve the growth‐maintenance trade‐off in rice
Source: Plant J. 2026 Oct 30;128(3):e71185. doi: 10.1111/tpj.71185 (PMC13722830; doi:10.1111/tpj.71185)
Supplement: Supplementary file 1 [file TPJ-128-0-s001.zip › Figures S1-S8 and Table S2.docx]

**Supporting information**

**Figure S1. The intact OsEF1Bγ interacts with all domains of OsYchF1.** (A and B) Bimolecular fluorescence complementation (BiFC) assays demonstrate the *in vivo* interaction between OsEF1Bγ-nGFP and truncated versions of OsYchF1, including OsYchF1 G domain-cGFP, OsYchF1 Helical domain-cGFP and OsYchF1 TGS domain-cGFP in *N. benthamiana* leaf epidermal cells (A) and rice leaf protoplasts (B). Scale bars = 10 µm. Cells were imaged using confocal microscopy.

**Figure S2. Structure-based alignment of OsEF1Bγ using software Jalview version 1.6.** This shows the structure-based alignment of OsEF1Bγ (NP_001389794.1), OsEF1Bγ-1 (BAA34206.1), and OsEF1Bγ-3 (BAD61828.1), *Arabidopsis thaliana* EF1Bγ (NP_563848.1, NP_176084.1) and, *Saccharomyces cerevisiae* EF1Bγ (NP_015277.1, NP_012842.1). Identical amino acid residues are marked in dark blue and homologous residues in light blue. Gaps are marked as dashes. α-helices are indicated by red lines and β-strands by green lines. The predicted binding residues (Asn-11 and Lys-12) are marked with asterisks.

**Figure S3. Molecular phylogenetic analysis of OsEF1Bγ and its homologues.** OsEF1Bγ, OsEF1Bγ-1, OsEF1Bγ-3, AtEF1Bγ, and ScEF1Bγ were analyzed by the Maximum Likelihood method. The protein accession numbers are as follow: OsEF1Bγ (NP_001389794.1), OsEF1Bγ-1 (BAA34206.1.1), OsEF1Bγ-3 (BAD61828.1), AtEF1Bγ (NP_563848.1, NP_001031202.1), ScEF1Bγ (NP_015277.1, NP_012842.1). Bootstrap values are recorded at the nodes.

**Figure S4. Sequence analysis of CRISPR-Cas9-mediated genome editing of *OsYchF1* in rice.** (A) The homozygous mutant *osychf1-1* carries a single‑nucleotide insertion of an ‘A’ relative to the reference sequence, which is present in both Allele1 and Allele2. (B) The *osychf1-3* mutant carries a homozygous deletion mutation. Both alleles show a nucleotide deletion “GTCG” relative to the reference sequence. (C) The homozygous mutant *osychf1-5* carries a single-nucleotide insertion of an ‘A’ relative to the reference sequence, which is present in both Allele1 and Allele2. For each mutant, the anchor and degenerate sequences used for genotyping are indicated above the alignments. Sequence alignments compare the mutant alleles with the reference sequence; dashes denote gaps.

**Figure S5. SDS-polyacrylamide gel electrophoresis analysis of the purified proteins.** Eluted fractions are shown in lanes 1, 2, and 3 for OsEF1Bγ, OsEF1Bγ (N11A, K12A), and OsYchF1, respectively. The predicted molecular masses of the His-SUMO-tagged OsEF1Bγ proteins (both wild-type and the N11A, K12A mutant) and OsYchF1 are 65.4 kDa and 44.3 kDa, respectively. M, protein marker.

**Figure S6. GST and antioxidant enzyme activity in the leaves of the WT, *OsEF1Bγ* overexpression and *OsYchF1* knockout mutant plants under normal and stressful conditions.** (A) GST activity in the leaves of WT, plants overexpressing *OsEF1Bγ* (*OsEF1Bγ* OE#1) and *OsYchF1* knockout mutants (*osychf1-1* and *osychf1-5*) plants under normal conditions and under osmotic stress induced by 15% PEG6000 or salt stress induced by 100 mM NaCl. (B) Catalase (CAT) activity in the leaves of WT and *OsEF1Bγ* overexpression (*OsEF1Bγ* OE#1 and 3) under normal conditions, and under 15% PEG6000-induced osmotic stress or 100 mM NaCl salt stress. (C) CAT activity in the leaves of WT and *OsYchF1* knockout mutants (*osychf1-1, osychf1-5*) under normal conditions, and under 15% PEG6000-induced osmotic stress or 100 mM NaCl salt stress. (D) Peroxidase (POD) activity in the leaves of WT and *OsEF1Bγ* overexpression (*OsEF1Bγ* OE#1 and 5) under normal conditions, and under 15% PEG6000-induced osmotic stress or 100 mM NaCl salt stress. (E) POD activity in the leaves of WT and *OsYchF1* knockout mutants (*osychf1-1* and *osychf1-5*) under normal conditions, and under 15% PEG6000-induced osmotic stress or 100 mM NaCl salt stress. (F) Superoxide dismutase (SOD) activity in the leaves of WT and *OsEF1Bγ* overexpression (*OsEF1Bγ* OE#1 and 5) under normal conditions, and under 15% PEG6000-induced osmotic stress or 100 mM NaCl salt stress. (G) SOD activity in the leaves of WT and *OsYchF1* knockout mutants (*osychf1-1* and *osychf1-5*) under normal conditions, and under 15% PEG6000-induced osmotic stress or 100 mM NaCl salt stress. Error bars indicate ±SD (n = 3 in three independent experiments). Values with the same letters are not significantly different from one another (one-way ANOVA, *p*<0.05).

**Figure S7. The GST inhibitor abolished the stress tolerance conferred by the *OsEF1Bγ* overexpression and *OsYchF1* knockout under stressful conditions.** (A) Representative phenotypes of eight-week-old WT Nipponbare (Nip), a transgenic line overexpressing *OsEF1Bγ* (*OsEF1Bγ* OE#1) and a CRISPR-Cas9-based gene-editing transgenic rice line (*osychf1-1*) with Nip as the background are shown under normal conditions, after treatment with 15% PEG6000 to induce osmotic stress and after treatment with 100 mM NaCl. (B) The plant length of eight-week-old WT, *OsEF1Bγ* OE#1 and *osychf1-1* plants under normal conditions. Plant length of eight-week-old WT, *OsEF1Bγ* OE#1 and *Osychf1-1* plants with 200 µM ethacrynic acid (EA) is shown in (C) when subjected to osmotic stress caused by 15% PEG6000, and in (D) after 100 mM NaCl treatment. (E) Shoot and root dry weight of eight-week-old WT, *OsEF1Bγ* OE#1 and *Osychf1-1* plants under normal conditions. Shoot and root dry weight of 8-week-old WT, *OsEF1Bγ* OE#1 and *Osychf1-1* plants with 200 µM EA upon osmotic stress caused by 15% PEG6000 (F), and after 100 mM NaCl treatment (G). Scale bars = 10 cm. Error bars indicate the SD; n≥5, and each experiment was repeated at least three times. Different letters above the bars indicate statistically significant differences (p < 0.05) as determined by the Tukey's HSD test.

**Figure S8. Structure-based alignment of OsYchF1 with its plant orthologs using Jalview software (version 1.6).** gi/138637314 is OsYchF1 in rice, gi/242080845 is *Sorghum bicolor* YchF, gi/225444063 is *Vitis vinifera* YchF, gi/255564375 is *Ricinus communis* YchF, gi/15221444 is *Arabidopsis thaliana* YchF, gi/116789076 is *Picea sitchensis* YchF, gi/168048097 is *Physcomitrella patens* YchF. α-helices (red lines) and β-strands (green lines) are indicated below the sequences. Evolutionarily conserved cysteine residues are marked with red asterisks.

**Table S2. Primers used in the study.**

Primer name	**Sequence 5’→3’**

OsYchF1-nLUC-F	CCGGTACCCATGCCGCCCAAGGCG

OsYchF1-nLUC-R	GTCGACCTT CTT TCCACC TCC AGAC

OsYchF1-G-nLUC-F	CC GGTACCC ATGCCGCCCAAGGCG

OsYchF1-G-nLUC-R	AAAGTCGAC TGCTCTCAATACATGAAAAATTCCATCAAC

OsYchF1-colied-nLUC-F CC GGTACCC ATGGTGGATCCTGTTAGAGATTTGGAAACTATT

OsYchF1-colied-nLUC-R AAAGTCGAC CTTTCCATCTTCAAGATGGGCTTT

OsYchF1-TGS-nLUC-F	CC GGTACCC ATGCTTATATACTTTTTCACTGCTGGCCCT

OsYchF1-TGS-nLUC-R	AAAGTCGAC CTTCTTTCCACCTCCAGACAC

OsYchF1-No TGS-nLUC-F CC GGTACCC ATGCCGCCCAAGGCG

OsYchF1-No TGS-nLUC-R GTCGAC ATG GAT TGC TGC AAAA CCA GTC

OsEF1Bγ-ccLUC -F	GGTACCATGGCGCTTGTGTTGCATTGC

OsEF1Bγ-N-ccLUC-R	GTCGACATTGCTTGAAATCACCAATGACCTTC

OsEF1Bγ-C-ccLUC-F	GGTACCAAACCAAAGGTTGAGGCTTCAG

OsEF1Bγ-ccLUC-R	GTCGACCTTGAAGCATTTTGCATCCAGCA

cLUC-OsEF1Bγ-F	GGTACC ATGGCGCTTGTGTTGCATTGC

cLUC-OsEF1Bγ-R	GTCGAC TCACTTGAAGCATTTTGCATCCAGC

pHBT-OsEF1Bγ-cYFP-F	GTCGAC ATGGCGCTTGTGTTGCATTGC

pHBT-OsEF1Bγ -cYFP-R	GGTACC CTTGAAGCATTTTGCATCCAGCA

pHBT-EF-NR-cYFP	GGTACC TTGCTTGAAATCACCAATGACCTTC

pHBT-EF-CF-cYFP	GTCGAC AAACCAAAGGTTGAGGCTTCAG

PHBT-OsYchF1 G-nYFP-F	CCGTCGACATGCCGCCCAAGGCG

PHBT-OsYchF1 G-nYFP-R	GGTACCTGCTCTCAATACATGAAAAATTCCA TCAACA

PHBT-OsYchF1-colied-nYFP-F CCGTCGACATGGTGGATCCTGTTAGAGATTT GGAAACTATT

PHBT-OsYchF1-colied-nYFP-R GGTACCCTTTCCATCTTCAAGATGGGCTTT

PHBT-OsYchF1 TGS-nYFP-F	CCGTCGACATGCTTATATACTTTTTCACTGCT GGCCCT

PHBT-OsYchF1 TGS-nYFP-R	GGTACCCTTCTTTCCACCTCCAGACAC

PHBT-OsYchF1 No TGS-nYFP -F CCGTCGACATGCCGCCCAAGGCG

PHBT-OsYchF1 No TGS-nYFP -R GGTACCATGGATTGCTGCAAAACCAGTC

qPCR-OsYchF1-F	ATGGTGGATCCTGTTAGAGATTTGGAAAC

qPCR-OsYchF1-R	CTTTCCATCTTCAAGATGGGCTTTGAC

qPCR-OsEF1Bγ-F	GATGAACAAGGTCGGTGGGT

qPCR-OsEF1Bγ-R	GGTCAACCTTGGTCCACTCA

His-OsYchF1-F	AAGAATTCATGCCGCCCAAGGCGTC

His-OsYchF1-R	CCCAAGCTTCTTCTTTCCACCTCCAG

His-OsEF1Bγ-F	ACCGGTGCCGCCGCCATGGCGCTTGTGTTGCATTGC

His-OsEF1Bγ-R	GAATTCTCACTTGAAGCATTTTGCATCCAGC
